# Visual-Tactile Speech Perception and the Autism Quotient

**DOI:** 10.3389/fcomm.2018.00061

**Published:** 2019-01-07

**Authors:** Donald Derrick, Katie Bicevskis, Bryan Gick

**Affiliations:** 1 New Zealand Institute of Language, Brain, and Behaviour, University of Canterbury, Christchurch, New Zealand,; 2 MARCS Institute, Western Sydney University, Milperra, NSW, Australia,; 3 School of Languages and Linguistics, University of Melbourne, Parkville, VIC, Australia,; 4 Department of Linguistics, University of British Columbia, Vancouver, BC, Canada,; 5 Haskins Laboratories, New Haven, CT, United States

**Keywords:** speech perception, multisensory speech perception, multimodal speech perception, audio-tactile perception, autism spectrum disorders

## Abstract

Multisensory information is integrated asymmetrically in speech perception: An audio signal can follow video by 240ms, but can precede video by only 60ms, without disrupting the sense of synchronicity (Munhall et al., 1996). Similarly, air flow can follow either audio (Gick et al., 2010) or video (Bicevskis et al., 2016) by a much larger margin than it can precede either while remaining perceptually synchronous. These asymmetric windows of integration have been attributed to the physical properties of the signals; light travels faster than sound (Munhall et al., 1996), and sound travels faster than air flow (Gick et al., 2010). Perceptual windows of integration narrow during development (Hillock-Dunn and Wallace, 2012), but remain wider among people with autism (Wallace and Stevenson, 2014). Here we show that, even among neurotypical adult perceivers, visual-tactile windows of integration are wider and flatter the higher the participant’s Autism Quotient (AQ) (Baron-Cohen et al., 2001), a self-report measure of autistic traits. As “pa” is produced with a tiny burst of aspiration (Derrick et al., 2009), we applied light and inaudible air puffs to participants’ necks while they watched silent videos of a person saying “ba” or “pa,” with puffs presented both synchronously and at varying degrees of asynchrony relative to the recorded plosive release burst, which itself is time-aligned to visible lip opening. All syllables seen along with cutaneous air puffs were more likely to be perceived as “pa.” Syllables were perceived as “pa” most often when the air puff occurred 50–100ms after lip opening, with decaying probability as asynchrony increased. Integration was less dependent on time-alignment the higher the participant’s AQ. Perceivers integrate event-relevant tactile information in visual speech perception with greater reliance upon event-related accuracy the more they self-describe as neurotypical, supporting the Happé and Frith (2006) weak coherence account of autism spectrum disorder (ASD).

## INTRODUCTION

Multisensory integration of speech information is evident in audio-visual (Sumby and Pollack, 1954; McGurk and MacDonald, 1976), audio-tactile (Gick and Derrick, 2009; Derrick and Gick, 2013), and visual-tactile (Bicevskis et al., 2016) combinations. For all three combinations, the temporal window of integration, where multisensory stimuli jointly influence perception, is asymmetric in a direction consistent with the relative speeds of the various signals (Munhall et al., 1996; Gick et al., 2010). Specifically, an audio signal can follow video by as much as 240ms and still be perceived as synchronous, but audio can only precede video by 60ms (Munhall et al., 1996) while still being perceived as synchronous; similarly, air flow can follow audio by as much as 200ms, but precede audio by only 50ms (Gick et al., 2010); in visual-tactile (non-auditory) speech perception, the air flow can follow the video by 300ms, but precede video by only 100ms (Bicevskis et al., 2016).

The width of these temporal windows of integration also varies across speakers. While the famous “McGurk effect” (McGurk and MacDonald, 1976), where individuals perceive a visual “ga,” and auditory “ba” as “da,” is highly stable, other syllable combinations are not as stable. For these other combinations, the closer in time an audio beep and visual flash need to be presented for a perceiver to consider them as coming from one source, the more likely that same perceiver is to experience McGurk effects (Stevenson et al., 2012). While there remain idiosyncratic reasons for these differences among perceivers, two general trends have been observed. These windows of integration narrow during childhood development (Hillock-Dunn and Wallace, 2012), but they remain broader among people with Autism Spectrum Disorder (ASD) (Wallace and Stevenson, 2014).

### Autism Spectrum Disorder

Research into ASD and typically developing (TD) child populations has found that children with ASD have poorer audiovisual temporal acuity (Bebko et al., 2006; Foss-Feig et al., 2010; Kwakye et al., 2011; Stevenson et al., 2014). Children with ASD also show less integration of audiovisual speech information as compared to neurotypical children (de Gelder et al., 1991; Mongillo et al., 2008), and are less likely show the “McGurk effect” (Mongillo et al., 2008). That is, children with ASD were significantly less likely to report the integrated speech sound, and more likely to report the audio stimulus, as compared to TD children. However, Taylor et al. (2010), found that any difference in rates of integration between the two populations disappeared by adulthood. This lack of integration in childhood may be at least in part due to poor speech reading skills (e.g., Williams et al., 2004) and deficits in facial identity and expression recognition in ASD populations (Davies et al., 1994). The observations hold true even when the children with ASD are known, through eye tracking, to be looking at the visual speech stimuli in question (Irwin et al., 2011). However, Stevenson et al. (2014) reported no significant differences between ASD and TD populations in visual-only speech categorization tasks. They suggest that the lower rate of McGurk percepts in the ASD population may have been due to difficulties integrating two stimuli from any modalities.

These observations are consistent with the “weak coherence” account (Frith, 1989; Happé and Frith, 2006); in typically developing individuals, low-level information components are combined to form higher-level meaning (global processing), but in individuals with ASD, information processing is thought to be characterized by a focus on these component parts (local processing). Weak coherence often results in exceptional skill when local processing is called for, but deficits related to processing global meaning. This account is therefore able to explain why individuals with ASD sometimes have very high skills related to uni-modal processing.

In contrast, the “theory of mind” account (Baron-Cohen et al., 1985; Baron-Cohen, 1995), which proposes that individuals with ASD have a diminished theory of mind causing difficulties in relating to others, does not account for the difference in multimodal processing between neurotypical and ASD populations. Nor does “executive dysfunction” theory (see e.g., Rumsey and Hamburger, 1988; Ozonoff et al., 1991), where executive function – a term that encompasses behaviors used for activities such as planning, organizing and regulating behavior – is thought to be damaged in individuals with ASD.

Instead, this body of multisensory research indicates that the more accurately people temporally align audio and visual speech information, the more their perceptions may be influenced by information from both of these modalities, and that people with ASD have reduced audio-visual speech alignment accuracy. These results leave open the question of whether this phenomenon extends to other modality combinations, (e.g., visual-tactile) and also whether there is a relationship between speech signal alignment accuracy and ASD that extends into the adult neurotypical population. These are questions we aim to address in the current study. To do this, we need a measure that relates features of the autism spectrum to the neurotypical population – the Autism Quotient (Baron-Cohen et al., 2001).

### The Autism Quotient

The Autism Quotient (AQ) questionnaire is a self-report test that measures traits in adults, certain levels of which are typically associated with autism. The test questions comprise five categories: (1) social skills, (2) communication skills, (3) imagination, (4) attention to detail, and (5) attention switching/focus of attention. The answers are converted into a formula – the AQ score – with higher scores representing higher levels of traits associated with ASD, such that 80% of adults with ASD score higher than 32 points, compared to 2% of the control population (Baron-Cohen et al., 2001). Note that Woodbury- Smith et al. (2005) later argued that the cut-off point should be lowered, finding that 83% of adults with ASD scored above 26 points.

The test has been translated into Dutch and Japanese, and all versions have a high test-retest value ranging from 0.63 to 0.78 (Hoekstra et al., 2008), with similar patterns whereby men tend to score higher than women, and scientists/science students higher than non-scientists (Baron-Cohen et al., 2001; Woodbury-Smith et al., 2005; Hoekstra et al., 2008). The consistency and repeatability of the AQ make it a useful tool in analysis of the potential factors that influence participant variance in temporal windows of integration. However, the nature of this data requires the use of careful and robust analysis.

### Present Study

**FIGURE 1.**
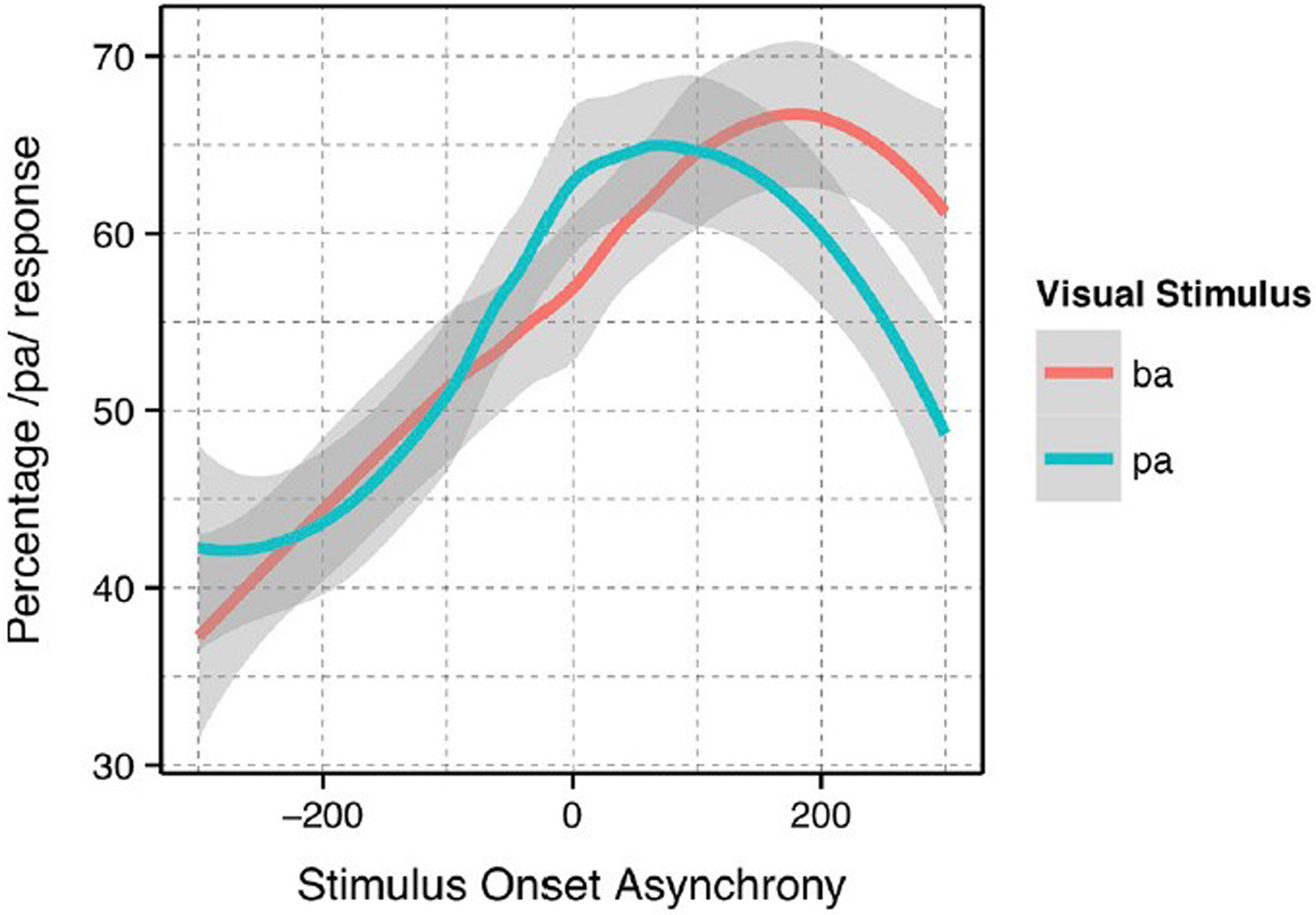
Summary results reproduced from Bicevskis et al. (2016) with the permission of the Acoustical Society of America.

The analysis presented was completed using previously collected data (Bicevskis et al., 2016). Again, the Bicevskis et al. study demonstrated that visual-tactile integration in speech perception will have an ecologically valid window of temporal integration much as audio-visual (Munhall et al., 1996; Gick et al., 2010) and audio-tactile (Gick et al., 2010) integration. That is, speakers are more likely to identify visual “pa” or “ba” as “pa” when they experience air flow on their skin at or after vowel onset, in the manner shown in Figure 1.

This re-analysis of that data was extended to compare the relationship between visual-tactile integration, the timing of visual and tactile stimuli, and participant Autism Quotient (AQ) scores. Visual-tactile stimuli were chosen because elimination of what is often considered the primary speech perception modality allowed for a more balanced relationship between the remaining sensory modalities—visual and tactile. The data included in this analysis were also expanded to include data collected on second language (L2) English participants.

#### Stimulus Onset Asynchrony

In addition, the method of identifying the asynchronicity for the stimulus onset asynchrony (SOA) was re-evaluated. In the original study, the SOA was calculated based on the point in time in which the artificial air flow stopped in relation to vowel onset. However, this measurement produced significantly different results for the underlying “pa” and “ba” data, as seen in Figure 1. The full description of our modifications to the SOA measurements are discussed in the methods.

### Hypothesis

Based on the findings of previous research, which showed that children with ASD exhibit poorer audiovisual temporal acuity (Bebko et al., 2006; Foss-Feig et al., 2010; Kwakye et al., 2011; Stevenson et al., 2014) and less audiovisual integration of speech information relative to typically developing children (de Gelder et al., 1991; Mongillo et al., 2008) and extending these findings to a new modality combination (visual-tactile), we make the following hypothesis: Neurotypical individuals who score higher on the AQ, i.e., self-report higher levels of traits associated with ASD, will integrate visual-tactile speech stimuli over a wider range of stimulus onset asynchronies (i.e., have flatter windows of visual-tactile integration) relative to those with lower AQ scores due to relatively poorer multimodal temporal acuity, and that they will exhibit lower rates of visual-tactile integration.

## MATERIALS AND METHODS

The current analysis is based on the relationship between data on visual-tactile speech perception, some of which was previously used to show asynchronous windows of integration for visual tactile speech (Bicevskis et al., 2016), and participants’ AQ scores.

### Materials

#### Video Stimuli

Video was recorded from one 28-year-old male native speaker of Vancouver English. He was instructed to produce eight natural repetitions of “pa” and “ba” in isolation. The productions were recorded on a model GZ-E300AU JVC camcorder, with 1,280 by 720 pixel resolution video at 24 frames per second and 48 kHz stereo PCM audio. Video was edited using Adobe Premiere ProCC. Five productions of each syllable (“pa” and “ba”) were chosen based on neutral facial expression, naturalness and consistency of production, and absence of eye-blinks. Each token was extracted, trimmed to 1,800ms so that the duration of each video was consistent, and saved as an individual file. Data were subjected to additive statistical analysis.

#### Synthetic Air Puffs

Puffs were generated using the same procedure as in Gick and Derrick’s original aero-tactile research (Gick and Derrick, 2009). The air compressor consisted of a 3-gallon (11.35-l) Jobmate oilless system connected to an IQ Valves on–off two-way solenoid valve (model W2-NC-L8PN-S078-MB-W6.0-V110) connected to a Campbell Hausfeld MP513810 air filter connected to J-inch vinyl tubing. The tubing was passed through a cable port into the soundproof room and mounted on a microphone boom-stand. The synthetic puff airflow was quickly turbulent upon leaving the tube, with an average turbulence duration of 100ms, slightly longer than the average voice onset time for “pa” (Lisker and Abramson, 1964) (see Figure 3 for one example of the underlying “pa,” which itself has a voice onset time longer than 100ms.) As with previous experiments, the output pressure of the synthesized puffs was adjusted to be minimally perceivable.

### Participants

This study was carried out in accordance with the Tri-Council Policy Statement guidelines of the University of British Columbia’s Behavioral Research Committee. The University of British Columbia’s Behavioral Research Committee approved the research protocol. Following the protocol, participants provided informed, written consent. In total, we tested fifty-five (55) participants. Five (5) participants were excluded due to experiment error: One participant altered the volume, the compressor was not turned on for two participants, and babble was not turned on for two more, leaving fifty (50) participants. These 50 participants were aged 18–40 years (μ = 21.1, s = 3.89, 10 male, 40 female). Thirty-one (31) were born to English speaking families, six (6) more were native speakers of Cantonese, three (3) Korean, four (4) Mandarin, one (1) Indonesian, one (1) Japanese, one (1) bilingual Cantonese-Mandarin, one (1) bilingual Japanese-Mandarin, one (1) Spanish, and one (1) Tagalog first-language (L1). All of these speakers learned English as a second language (L2) at the age listed in Table 1.

Three of these speaker’s native languages have no English-like “pa” vs. “ba” distinction. These were participant 11, whose L1 was Tagalog and learned English at age 2, participant 43 whose L1 was Japanese and learned English at age 3, and participant 44, whose L1 was Indonesian and learned English from age seven.

Participants reported no history of speech or hearing issues. They completed a demographic questionnaire and Autism Quotient (AQ) questionnaire. Participants were told they may feel air puffs on their skin during the experiment, but were otherwise uninformed as to the research design or goals.

### Procedure

Participants were seated within a sound-attenuated booth, with their head against a headrest. An air tube was positioned 7 cm from the front of their neck just above the sternum at the suprasternal notch. Participants wore Direct Sound EX29 headphones through which they heard continuous English multi-talker babble in order to mask the sound of air flow through the air tube. Participants were then given the two-alternative forced choice visual-tactile integration task, administered using custom software written in PsychoPy (Peirce, 2007). Participants were given instructions to watch the person on the screen speaking and respond via keyboard as to what he had said.

While listening to English multi-speaker babble and watching the silent videos of “pa” or “ba,” participants either received gentle puffs of air to their skin during trials or no air puff (control condition). The puffs of air were delivered at various SOAs, where SOA was defined as the time between the end of the air flow leaving the tube and the vowel onset from the original audiovisual recording (see Figure 3). The SOAs were 0ms (synchronous), ±50, ±100, ±200, and ±300ms, where “+” means that the visual stimulus precedes the tactile stimulus and “-” means the tactile stimulus precedes the visual. In addition, there was a control condition with no air flow at all.

**FIGURE 2.**
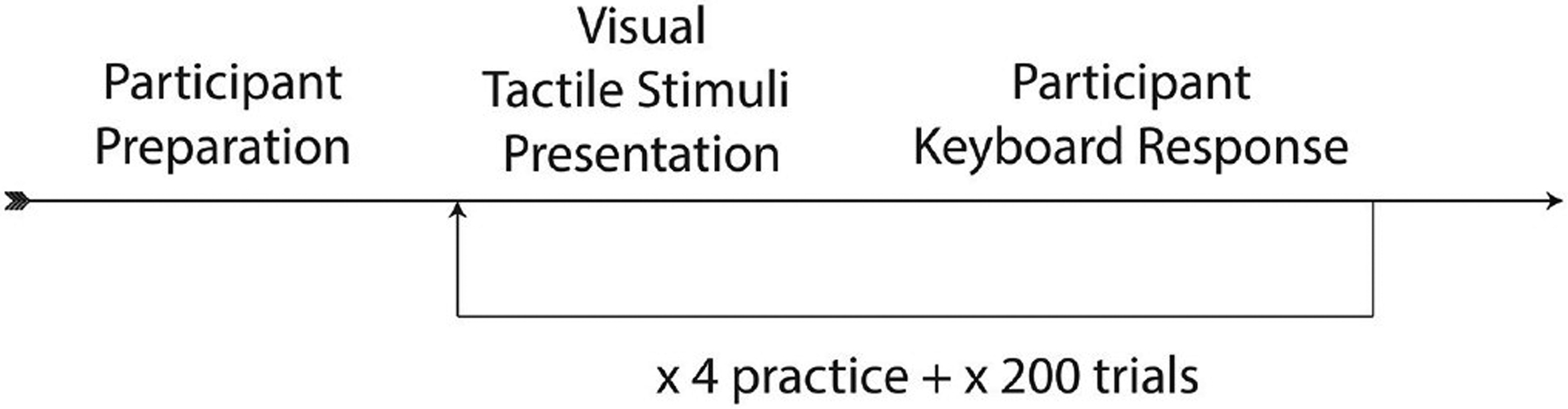
Paradigm of visual-tactile experiment.

The videos themselves included five silent video recordings each of “ba” and “pa.” The syllable “pa” and “ba” are considered visually indistinguishable (Fisher, 1968), though computers can detect subtle differences (Abel et al., 2011). Each condition was presented twice for each “pa” and “ba” visual stimulus, for a total of 180 tokens with air flow, and 20 tokens without air flow, or 200 experimental tokens. Tokens were presented in randomized order. Each participant watched 4 practice tokens, followed by the experimental tokens. Participants numbered 1–32 pressed the “z” key to indicate “ba” and the “/” key to indicate “pa,” and the rest of the participants (33–55) pressed the “/”-key to indicate “ba” and the “z” key to indicate “pa.” (The imbalance was due to an overestimation of the number of expected participants.) The paradigm can be seen in Figure 2. The task took <15min to complete. Since each participant experienced all the conditions, this experiment is a within-participant design.

### Stimulus Onset Asynchrony

**FIGURE 3.**
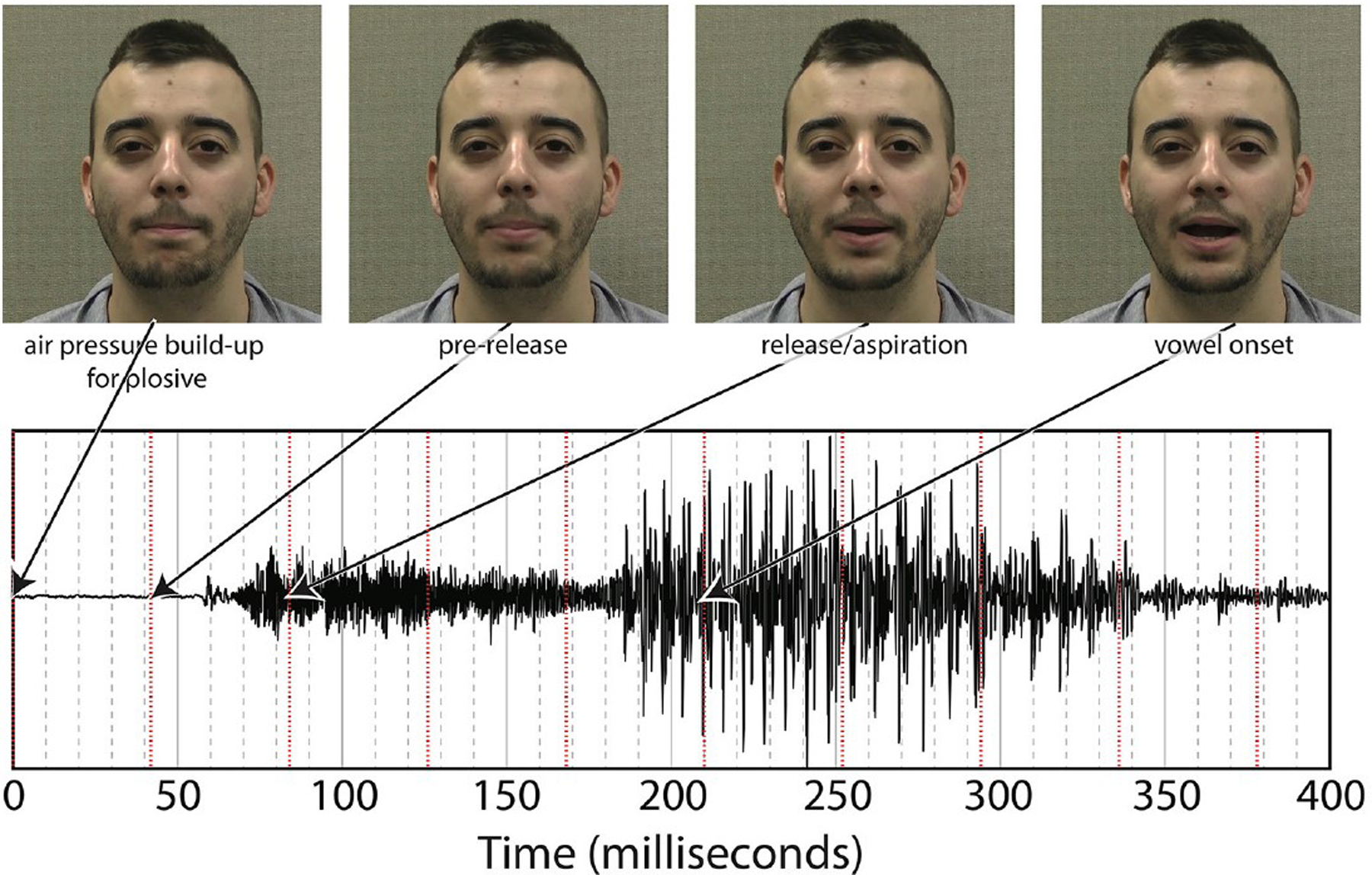
Relationship between underlying audio and facial motion for a token of “pa” (pa token 1).

As noted in the introduction, we decided to re-analyze the positioning of our SOA. The reason for doing so was based on the difference between the windows of integration between “ba” and “pa,” as seen in Figure 1. The reason for this difference might have been that one of the most salient features of the visual stimuli, lip opening, begins not with vowel onset, but with the onset of the plosive release burst, as seen in Figure 3.

**FIGURE 4.**
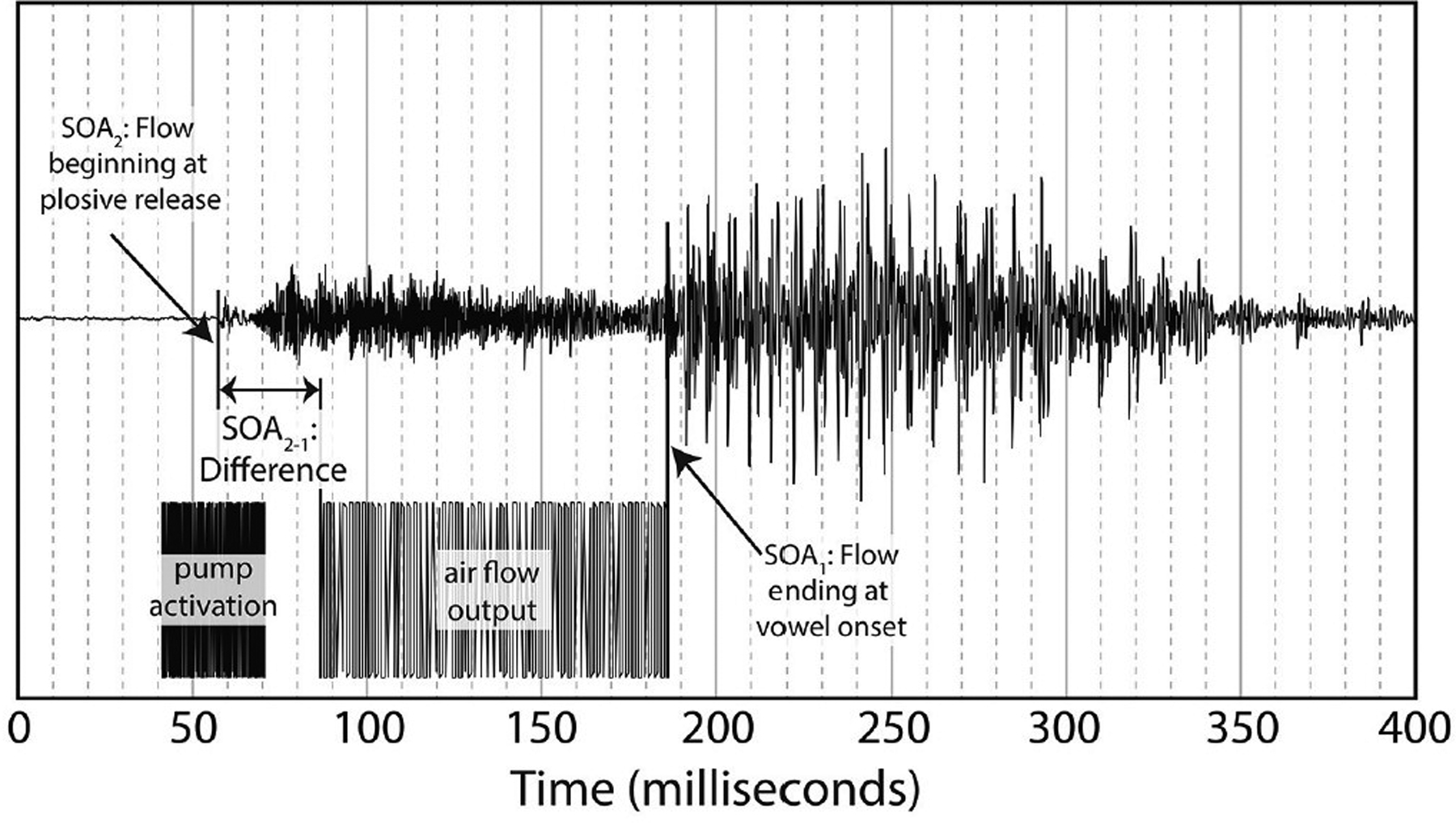
Difference between SOA as measured by end of air flow at vowel onset, and SOA as measured by beginning of air flow at plosive release. Example taken from the underlying acoustics of our video “pa,” token 1.

This difference is especially pronounced with “pa” data as there is a 100ms or greater delay from the lip opening at plosive release burst onset and the onset of vowel vocalization, as seen in Figure 4.

Figure 4 shows the methodology for the SOA difference calculation used in this analysis. This alternative method was then applied to each of the video tokens used in the original research, shifting each of the SOAs used in our final analysis. The new SOA displacement is shown in Table 2.

**FIGURE 5.**
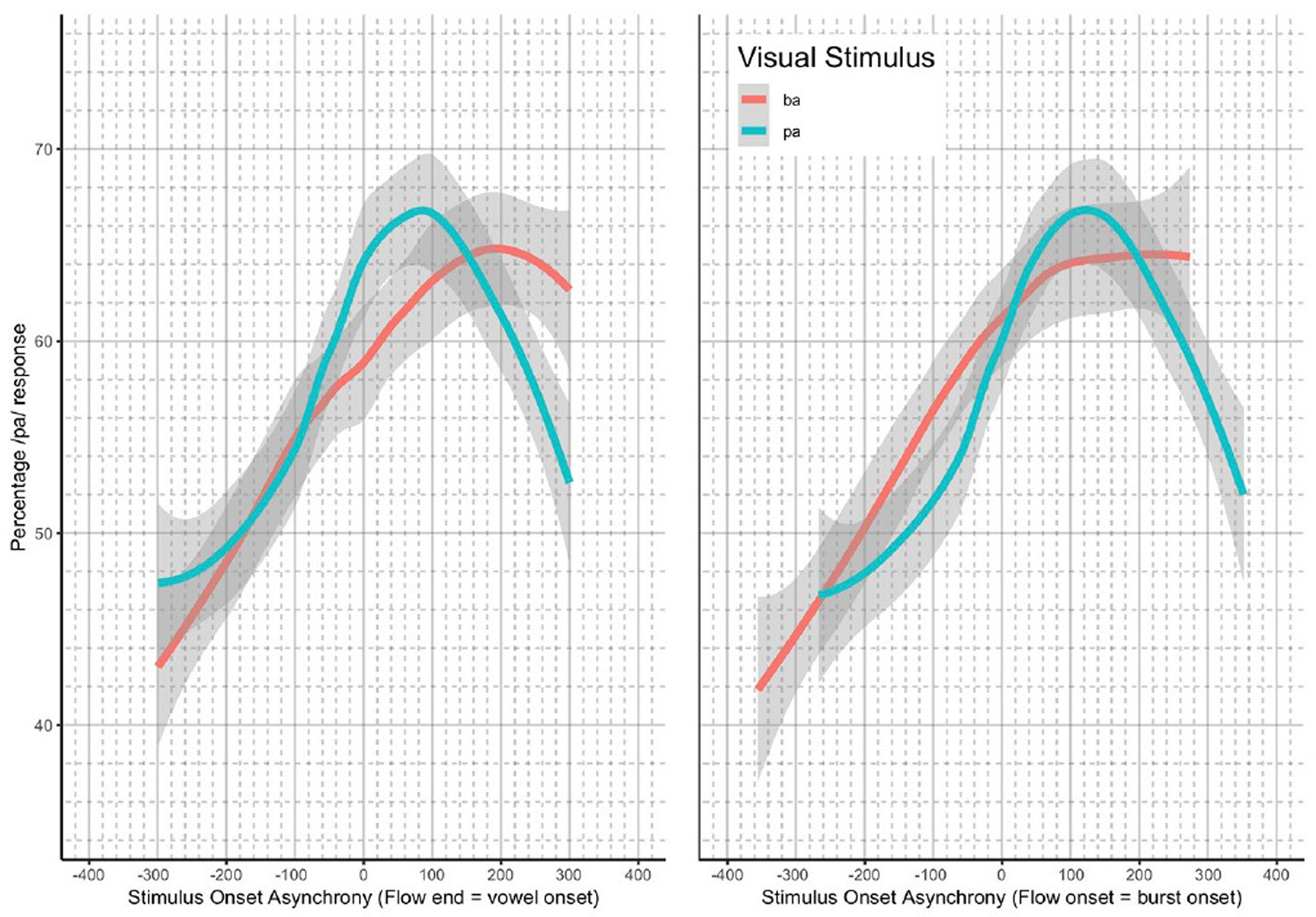
Loess and 95% confidence intervals of the windows of integration for all participants-SOA measurement methods compared.

Loess estimates and 95% confidence intervals of the windows of integration for all of the participants combined are shown in Figure 5. The Figure includes the loess estimates for SOA calculated from air flow ending at vowel onset, and the revised SOA estimate calculated from air flow onset aligned to burst onset. Figure 5 also shows that there are no gaps between the “ba” and “pa” estimate curves for the revised SOA measure. This reanalysis of SOA was used as part of the data analysis.

### Analysis

Descriptive statistics detailing the number of participants based on AQ score, and local polynomial fitting (loess) of their windows of integration by autism quotient were calculated. Generalized additive mixed-effects models (GAMM) (Wood, 2011) were then run in R (R Core Team, 2018) on the interaction between responses (“ba” or “pa”), SOA and AQ. GAMMs are particularly appropriate because they are non-linear statistics capable of using differences in window shape, or envelope, to identify significantly different patterns of behavior independent of overall changes. In this way, it is possible to separate out the relationship between AQ and overall integration rates as compared to the shape of the window of integration. Model fitting was then performed in a stepwise backwards iterative fashion; models were back-fit along the Akaike information criterion (AIC) to measure quality of fit. The Akaike information criterion (AIC) is an estimator of the comparative quality of statistical models, where the lower number represents a model with better fit. We used Maximum-likelihood where the AIC could not be used. Both back-fit tests were completed using CompareML method (van Rij et al., 2017). This technique allows for the isolation of a statistical model that provides the best fit for the data, and allows elimination of interactions in a statistically appropriate manner.

We also ran robusticity tests to ensure that that the influence of the two participants with the highest AQ scores, the influence of the L2 perceivers whose native languages did not include a “pa” vs. “ba” distinction, and the overall influence of L2 perceivers did not unduly disrupt or alter the patterns observed among the rest of the participants.

## RESULTS

### Descriptive Statistics

**FIGURE 6.**
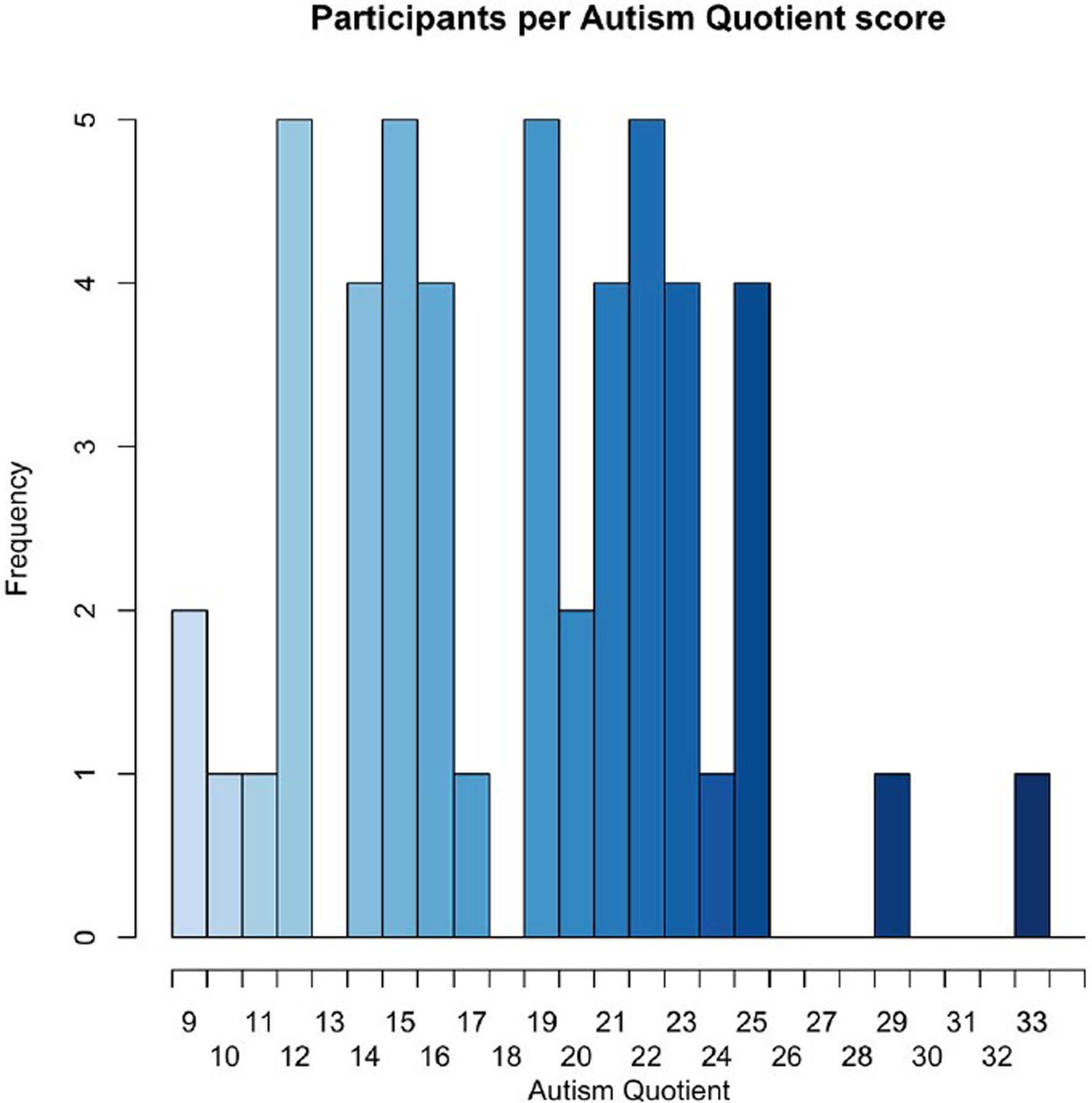
Histogram of participants by AQ score.

The fifty participants had a relatively even distribution of AQ scores, with the exception of two participants, one with an AQ of 29, and another with an AQ of 32, as seen in Figure 6.

**FIGURE 7.**
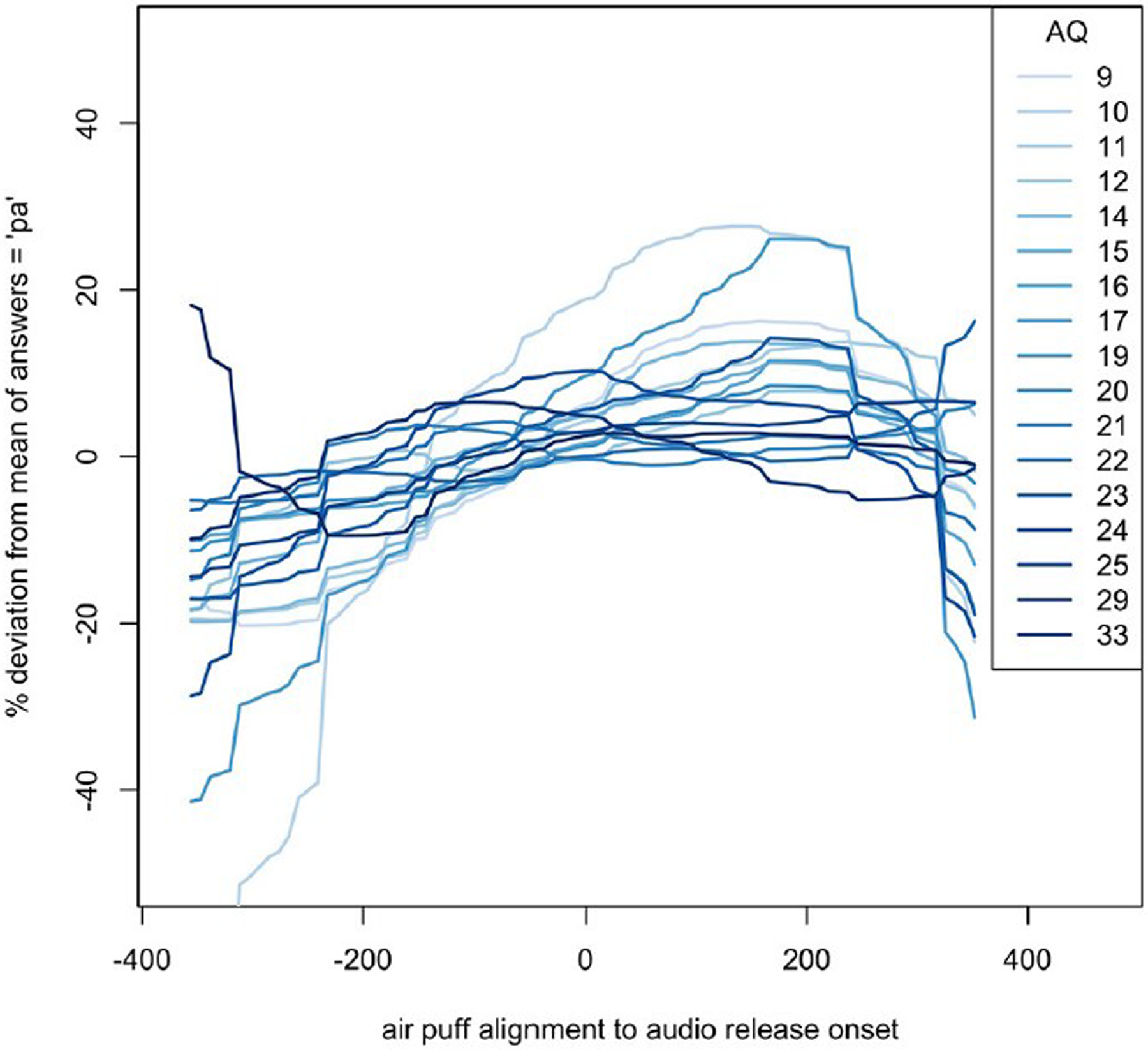
Loess of windows of integration for each AQ score, relative to their average (mean) responses. The SOAs shown are based on the new analysis described in the methods.

The windows of integration for each AQ score show that, in general, the higher the autism quotient, the flatter the response deviation – higher AQ participants did not alter their responses as much based on how far away in time air flow contact was fromthe lip opening. These estimates can be seen in Figure 7.

### Generalized Additive Mixed-Effects Models

In order to identify whether the general trend seen in the loess graphs in Figure 4 was statistically significant or not, a general additive mixed-effects (GAMM) model was applied to the data. After backwards iteration was applied to the model and all nonsignificant interactions were eliminated, following the methods described in (Sóskuthy, 2017), the final model was identified, and is shown in Equation 1.

Equation 1: GAMM test for comparing response to SOA and AQ.

response~s(SOA)+s(AQ)+s(Trialorder)+ti(SOA, AQ)+s(SOA, subject, bs=“re”)+s(AQ, subject, bs=“re”)+s(Trialorder, subject, bs=“fs”, m=1)+ti(SOA, AQ, subject, bs=“fs”,m=1)+s(token, bs=“re”)

*Response* is either 0 or 1, with 0 representing “ba” and 1 representing “pa.” The *SOA* term represents the Stimulus Onset Asynchrony. The *AQ* term represents the autism quotient. The *Trialorder* term represents the trial order. The *token* term represents the underlying video token. The first term *s(SOA)* is a spline-based *smoothing formula* for the main effect of SOA. These smoothing terms are similar to those used in smoothing-spline analysis of variance, where the curve is based on localized linear estimation. The second term *s(AQ)* is a spline-based smoothing formula for the main effect of autism quotient. The third term *s*(*Trialorder*) is a smoothing formula for the overall effects of trial order. The fourth term *ti*(*SOA*, *AQ*) is a tensor for the interaction between SOA and autism quotient. Tensors are a type of smoothing method that provide a stable an interpretable way of specifying models with main effects and interactions, allowing the s(SOA) and s(AQ) terms to carry all of the main effect information, leaving *ti*(*SOA*, *AQ*) to account for the interactions only – the shape of the window of integration. The fifth term *s*(*SOA*, *subject*, *bs* = “*re*”) is a random effect smoothing term for SOA by subject. The sixth term *s*(*AQ*, *subject*, *bs* = “*re*”) is a random effect smoothing term for AQ by subject. The seventh term *s*(*Trialorder*, *subject*, *bs* = “*fs*”, *m* = *1*) is an complex interaction smoothing term for trial order by subject. The eighth term *ti*(*SOA*, *AQ*, *subject*, *bs* = “*fs*”, *m* = *1*) is a complex interaction smoothing term for SOA and AQ by subject. The ninth and last term *s*(*token*, *bs* = “*re*”) is a simple smoothing term for each of the 10 tokens (5 of “ba” and 5 of “pa”). The model was run using the maximum-likelihood (ML) method. The results of the model can be seen in Table 3.

**FIGURE 8.**
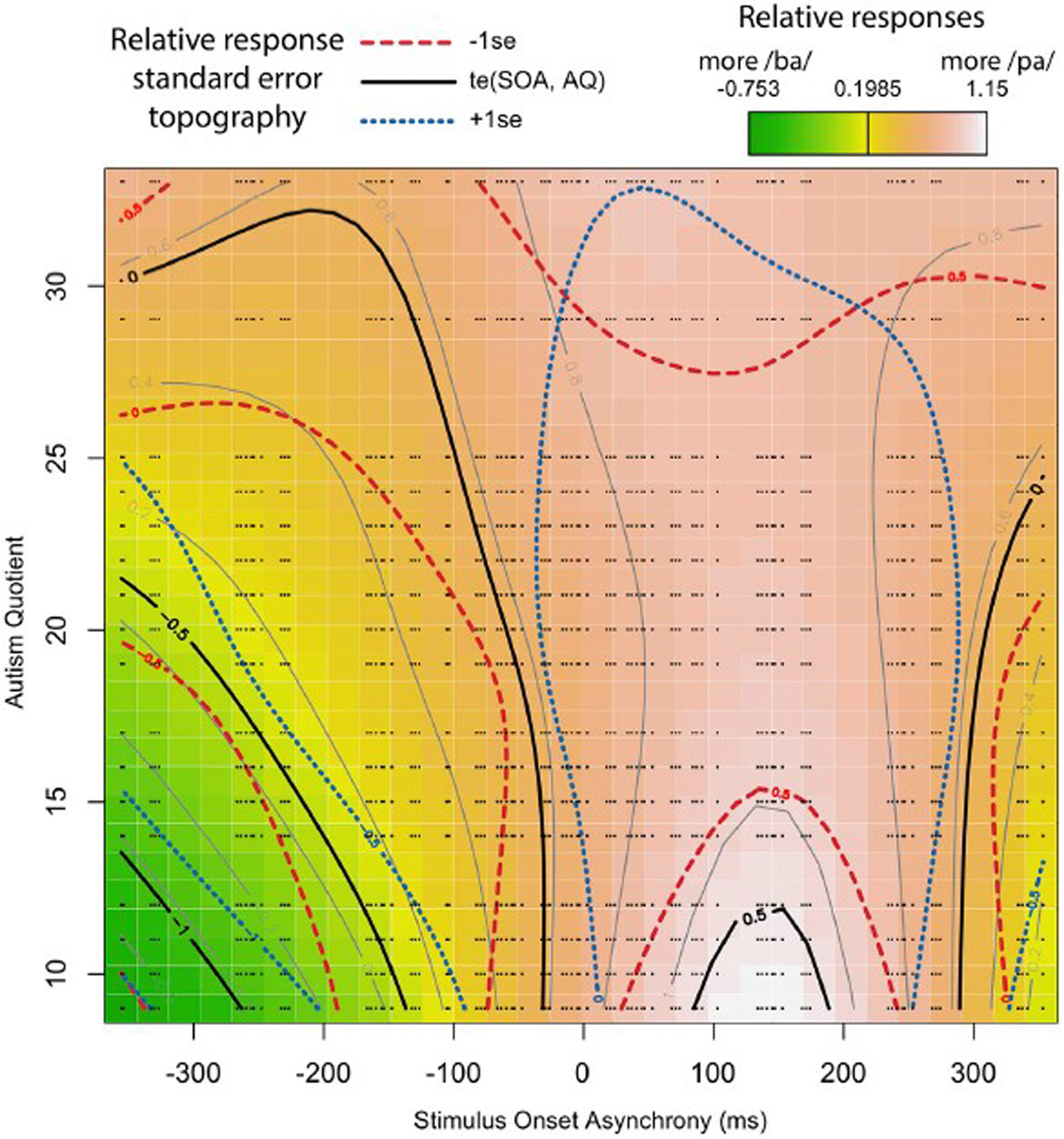
Interaction between SOA and AQ: Black lines represent a model-based smoothed arbitrary boundary of similar “pa” responses based on SOA and AQ. The red long-dashed lines represent -1 standard error from black lines. The blue short-dashed lines represent +1 standard error from black lines. The color map is a standard terrain map, with dark green representing more “ba” responses, and white representing the most “pa” responses. The small black dots represent the exact positions of recorded data points along SOA and AQ.

The adjusted R-squared is 0.176, explaining 16.5% of the deviance (Maximum Likelihood (ML) = 5.52 E 3, Number = 180 trials in the analysis * 50 participants = 9,000). The results show that the relationship between SOA and response is significant such that the participants differed in their reported perceptions based on SOA. In addition, there is also a significant relationship between SOA and AQ. The random effects of trial order, subject, and token are also highly significant. Interpreting these results is aided by visualization, as seen in Figure 8. Figure 8 is based on a tensor formula, which produces a less variegated image than the formula above, but allows standard error mapping. The use of standard error mapping helps in visualizing the nature of the significant relationships between SOA and autism quotient.

The results show that, for participants with a low AQ score, their temporal windows of integration are narrower, demonstrating greater temporal acuity in processing visual and tactile speech information. This shows up as steep color changes, similar to what one sees on topographical maps of steeply mountainous regions. As AQ increases, the shape of the temporal window of integration flattens. That is, the relationship between lip opening/facial relaxation and the timing of the air puff diminishes from the perspective of the perceiver. This shows up in the map as less steep changes in color across the SOA, similar to what one see on maps of flatter terrain. The overall impact is as looking at a mountain with the ridge along the SOA = 0 line, and whose peak is at the bottom of the map.

### Back-Fitting

It is not enough to show significant effects in GAMM models, we must also demonstrate that the relevant components of the model also account for significant portions of the variability in the overall model, and that the model is not missing necessary components. This is accomplished by back-fitting, or comparing the maximum-likelihood results in the GAMM equivalent of an F-test; several such comparisons support our model choice.

A linear variable testing for the relationship between AQ and likelihood of responding “pa” showed that people with higher AQs were significantly more likely to respond “pa,” but to such a small degree that including this term had no measurable effect on model variability. Following the standards of back-fitting, that term was eliminated from the final model. In addition, attempts to identify which of (Baron-Cohen et al.’s, 2001) original five categories were significant influences within the AQ yielded no significant results.

Comparison of the presented model to an otherwise identical model without the AQ included shows that the AQ accounts for a significant portion of the variability (p = 0.003, AIC = −7.17). Similarly, comparison of the presented model to an otherwise identical model but with no interaction between AQ and SOA shows the interaction alone also accounts for a significant portion of the variability (p < 0.001, AIC = −11.6).

In addition, comparison of the presented model to an identical one with the SOA calculated from vowel onset shows the model with SOA calculated from underlying release burst onset accounts for more of the variability (ML = 14.1, AIC = −28.4) (maximum-likelihood estimates, or ML, is here used when a p-value cannot be directly computed. It is similar to the AIC, where lower numbers represent better fitting models). In addition, a comparison of the presented model to an identical one that adds in a factor accounting for a group difference between underlying “ba” and “pa” videos provides no significant reduction in model variability (p = 0.756, AIC = −0.060). Comparison of the presented model to an identical one that adds in a factor that includes the year the participants learned English shows no significant effect of the age when participants learned English (see Supplementary Materials).

### Robusticity Tests

We also ran all of these tests using the same models, but excluding (1) the two high AQ participants, (2) the three non-native speakers whose native language does not have a “ba” vs. “pa” distinction, and (3) all of non-native speakers. None of these eliminated any of the significant outcomes.

## DISCUSSION

Our findings support the hypothesis that there is a relationship between perceivers’ autism quotients and their visual-tactile windows of integration such that people who self-report higher levels of traits associated with ASD have wider and flatter windows of integration than those with lower levels of those same traits. This is indicative of these higher AQ individuals having poorer temporal acuity – integration is maintained at asynchronies considered separate events by individuals with lower AQs. In this sense, the current findings are in accordance with those of Bebko et al. (2006), Foss-Feig et al. (2010), Kwakye et al. (2011) and Stevenson et al. (2014), who found poorer temporal acuity in audiovisual processing in child ASD populations.

This study did not, however, find significantly lower rates of integration in individuals who self-reported higher levels of traits associated with ASD. Our findings therefore differ from those of de Gelder et al. (1991) and Mongillo et al. (2008) who found that children with ASD showed less integration of audiovisual speech information, and Mongillo et al. (2008) who found that children with ASD experienced less McGurk effect than neurotypical children.

Originally, the weak coherence account proposed that individuals with ASD have a deficit in global processing (Frith, 1989). More recently and in the face of more related research, the theory has been modified to propose that local, rather than global, processing in individuals with ASD is a bias rather than a deficit, and that global processing is possible when required (Happé and Frith, 2006). Happé and Frith also suggest that local vs. global processing may be ends of a continuum of cognitive style which is also present in a neurotypical population and that weak coherence in ASD populations is just one aspect of the “broader autism phenotype.” They extend the account to produce a more full map of multi-modal decoupling, whereby mild decoupling is a de-association of signals in time, and more severe decoupling involves a focus on one of the sensory modalities. Given the current findings of poorer temporal acuity in individuals who self-report higher levels of traits associated with ASD, but not lower rates of visual-tactile integration in those same individuals, these findings can be interpreted as consistent with this updated weak coherence account (Happé and Frith, 2006), where poorer temporal acuity might be expected to occur where there is a bias to focus on component parts, but where integration of information from both modalities to form a percept is still possible. It may also be the case that because the individuals with higher AQ scores in the present study do not have clinical ASD diagnoses, they are less likely to fail to integrate, though their ability to do this with temporal acuity is poorer.

In both comparisons, we must note the differences between the previous studies and the present one. In all the previous studies the stimuli presented were audiovisual, and only synchronous stimuli was used in McGurk tasks so we are unable to get a sense of the shape of individuals’ windows of temporal integration in the previous studies in the same way as we can in the present research. Further, the individuals under examination in the previous studies were children and all previous studies compared ASD and neurotypical child populations, whereas the present study looks at levels of traits associated with ASD as a continuum within a neurotypical adult population.

Considering these contrasts with previous studies, this study can be seen as an extension of work previously done in the area of multimodal processing in ASD populations. We find that, even in a neurotypical population, multimodal acuity is related to levels of traits associated with ASD. Taken along with the pre-existing body of research, we can view this behavior as extending from very low AQ perceivers who have temporally acute multi-modal integration, to higher AQ but otherwise neurotypical perceivers with more weak coherence, to ASD perceivers who focus on one of the sensory modalities of speech to the increasing exclusion of the others.

In this way, the features of ASD extend beyond the realm of psychological disorders to all of us. Note that Hoekstra et al. (2008) ran a factor analysis of all the questions on the Autism Quotient, using criteria independent of (Baron-Cohen et al.’s, 2001) original categories, using only the original questions, to see which questions have the most predictive power. They concluded that the AQ measures two main factors, which they called “social interactions” and “attention to detail.” While our attempts to identify if the original five listed categories of the AQ test were individually relevant – they were not – future research into the relative importance of Hoekstra’s two factors in relation to multisensory perception is warranted: Do all the features of ASD extend into the neurotypical population to influence speech perception, or just questions that relate to certain features of ASD?

The current study also extends findings relating ASD populations and audiovisual integration to other modalities, showing that a relationship between acuity in visual-tactile speech integration and ASD traits exists. Further, we find that this relationship is maintained into adulthood, in contrast to (Taylor et al., 2010) finding that the differences in audio-visual integration between ASD and TD child populations disappeared by adulthood.

This research is, however, just the beginning, and should be followed by continued research into the potential relationships between autistic traits in the neurotypical and ASD population and multi-modal speech classification and perception tasks. This includes tri-modal research in audio-visual-tactile speech, and research into effects on continuous speech perception.

## Supplementary Material

1_SOA_AQ.pdf

3_SOA.pdf

4_AQ.pdf

2_SOA_SE.pdf

5_trial_order.pdf

6_SOA_subject_quantile.pdf

output_GAMM.txt

7_AQ_subject_quantile.pdf

8_trial_order_subject.pdf

9_token_quantile.pdf

10_ACF.pdf

12_ACF_subject_2.pdf

11_ACF_subject_1.pdf

SOA_models_GAMM_AQ.R

all_participants_AQ_summary.csv

Guide to Supplementary Materials

## Figures and Tables

**TABLE 1 |:** L2 English participants by L1 and Age of English acquisition (years).

Participant number	L1	Age of English acquisition (years)
1	Korean	15
4	Cantonese	4
5	Mandarin	4
7	Japanese, Mandarin	10
9	Mandarin	9
11	Tagalog	2
12	Cantonese	2
14	Mandarin	6
21	Cantonese	13
26	Korean	7
27	Cantonese	6
31	Cantonese	7
36	Cantonese	3
42	Korean	9
43	Japanese	3
44	Indonesian	7
46	Mandarin	8
49	Cantonese, Mandarin	9
51	Spanish	8

**TABLE 2 |:** SOA differences, in milliseconds, for each video file used in the experiment.

Syllable	Token number	SOA_2−1_ difference (milliseconds)
“ba”	1	−30
	2	−27
	3	−54
	4	−56
	5	−33
“pa”	1	52
	2	36
	3	40
	4	33
	5	52

**TABLE 3 |:** Fixed and random effects of GAMM.

	Estimate	Std. error	*z*-value	*p*-value
(Intercept)	0.444 E 0	1.68 E-1	2.64 E 0	8.26 E-3**
	edf	Ref.df	Chi.sq	*p*-value
s(SOA)	4.64 E 0	5.65 E 0	1.12 E 2	<1.00 E-3***
s(AQ)	1.00 E 0	1.00 E 0	3.17 E-1	5.74 E-1
s(Trial order)	1.00 E 0	1.00 E 0	2.91 E 0	8.79 E-2.
ti(SOA* AQ)	3.24 E 0	4.07 E 0	1.38 E 1	8.84 E-3**
s(SOA, subject)	7.96 E 0	4.80 E 1	9.69 E 0	<1.00 E-3***
s(AQ, subject)	1.70 E-3	4.80 E 1	1.50 E-2	<1.00 E-3***
s(Trial order, subject)	1.87 E 2	4.48 E 2	1.05 E 3	<1.00 E-3***
ti(SOA, AQ, subject)	5.79 E 1	1.97 E 2	1.15 E 2	<1.00 E-3***
s(token)	8.70 E 0	9.00 E 0	2.55 E 2	<1.00 E-3***

edf, empirical distribution function; Ref.df, degrees of freedom;

*** p ≤ 1.00 E-3,

** p ≤ 1.00 E-2,

* p ≤ 5.00 E-2,

“.” P ≤ 1.00 E-1.
