# Supplementary figures and images for: Visual-Tactile Speech Perception and the Autism Quotient
Source: Front Commun (Lausanne). Author manuscript; Available in PMC 2022 Jan 31. (PMC8802876; doi:10.3389/fcomm.2018.00061)

# linear predictor

-0.753      0.1985      1.15

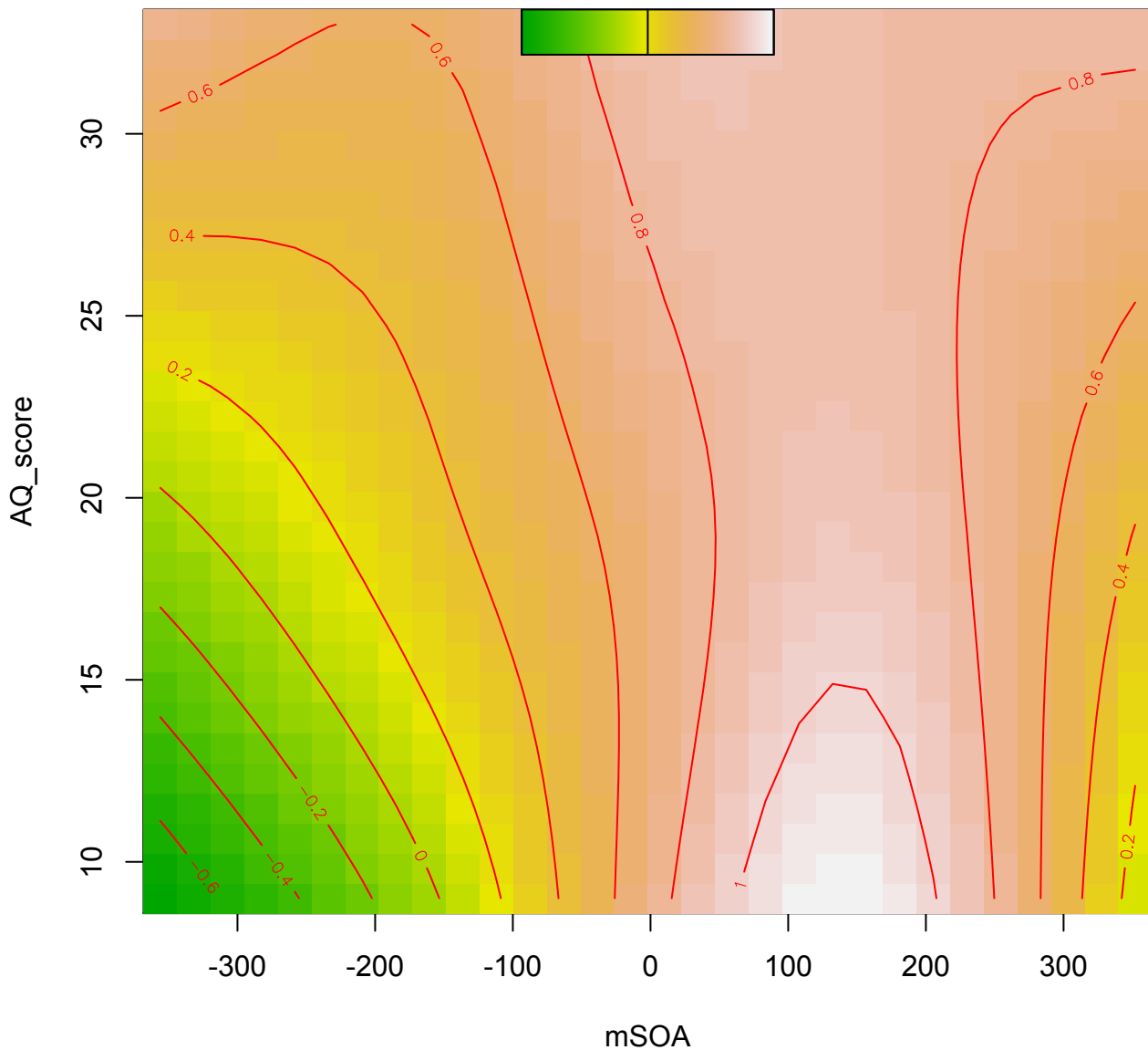

Supplement: 1_SOA_AQ.pdf [file NIHMS1725297-supplement-1_SOA_AQ_pdf.pdf]

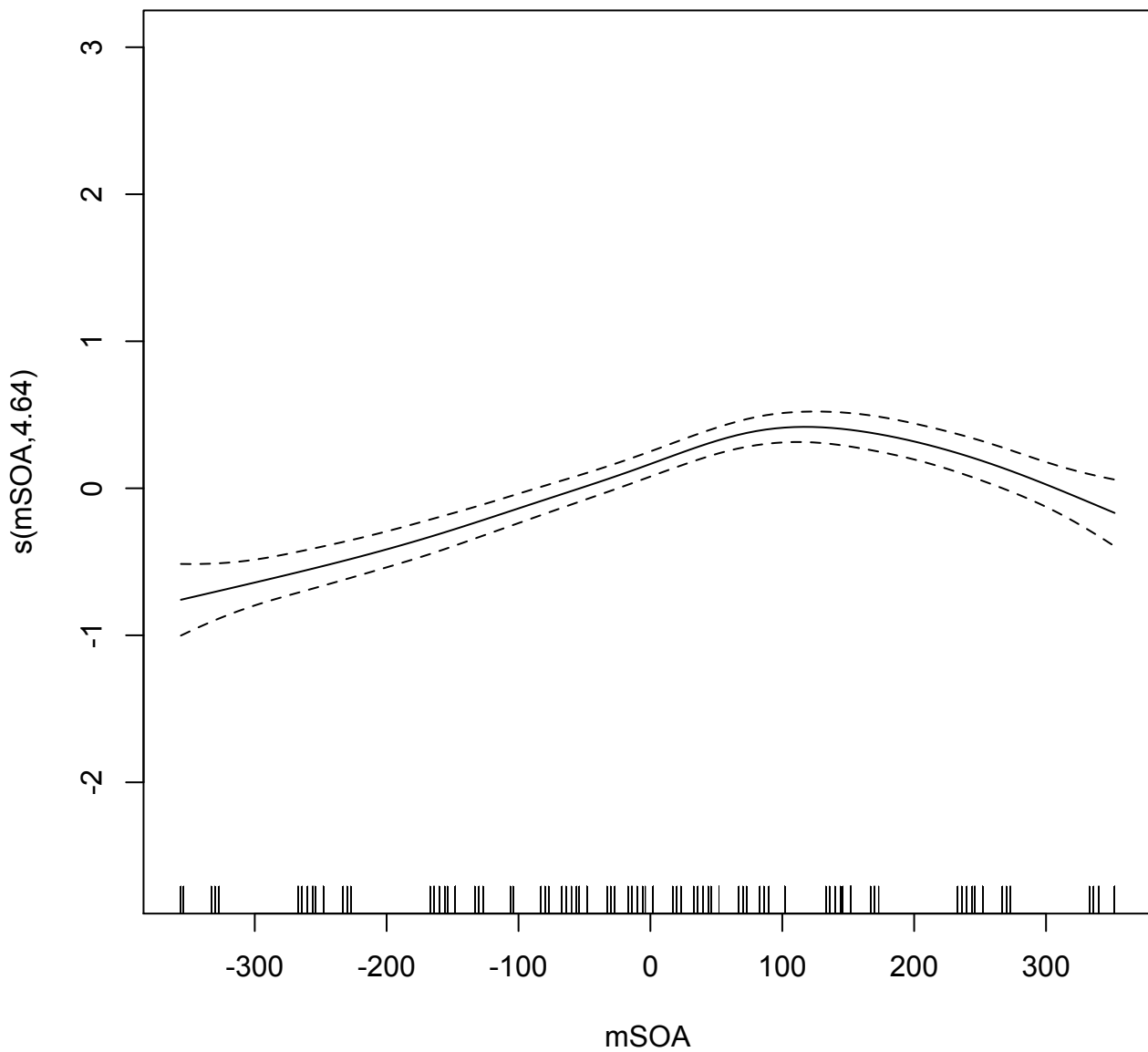

Supplement: 3_SOA.pdf [file NIHMS1725297-supplement-3_SOA_pdf.pdf]

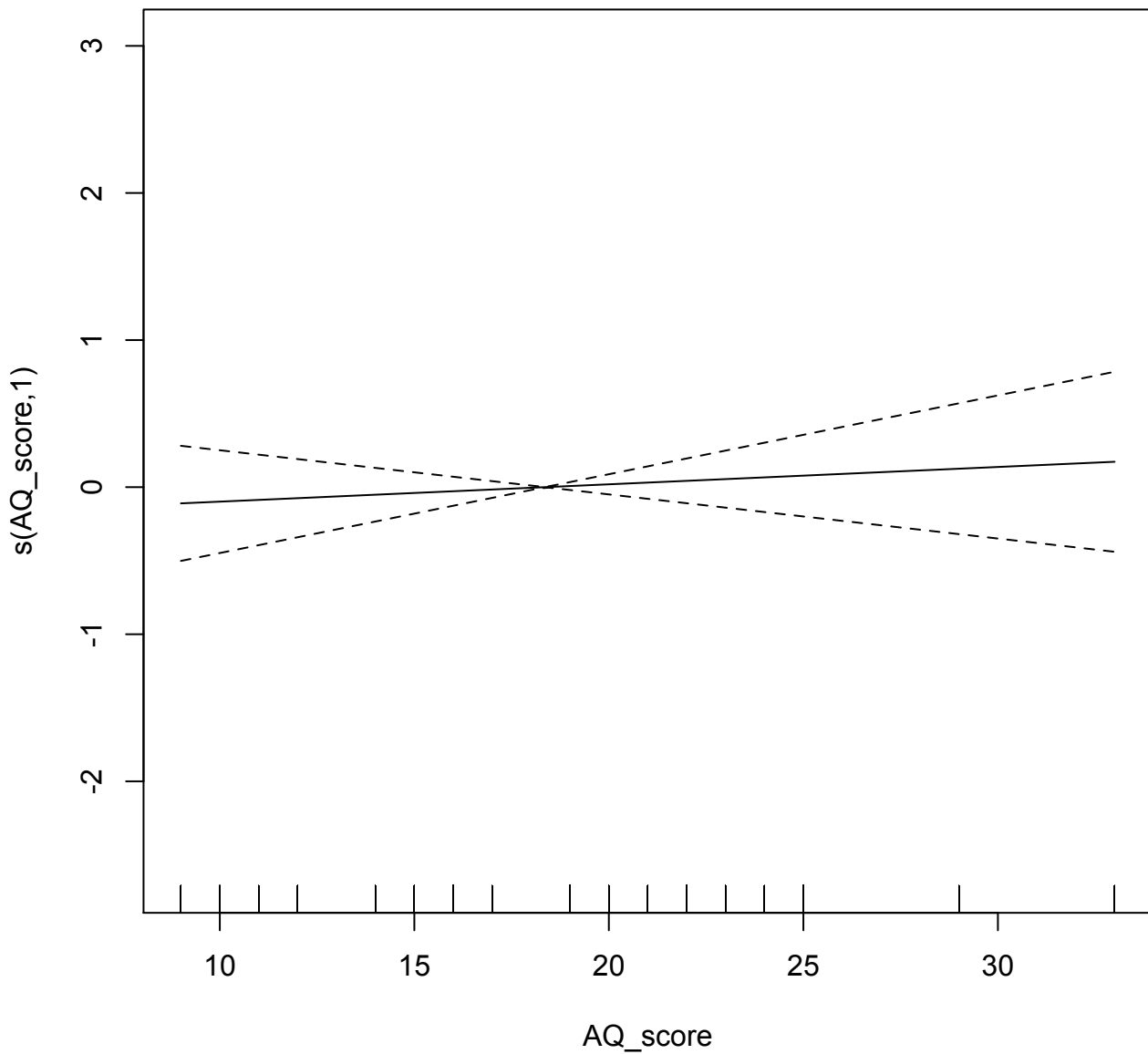

Supplement: 4_AQ.pdf [file NIHMS1725297-supplement-4_AQ_pdf.pdf]

Autism Quotient

-1se

te(SOA,AQ)

+1se

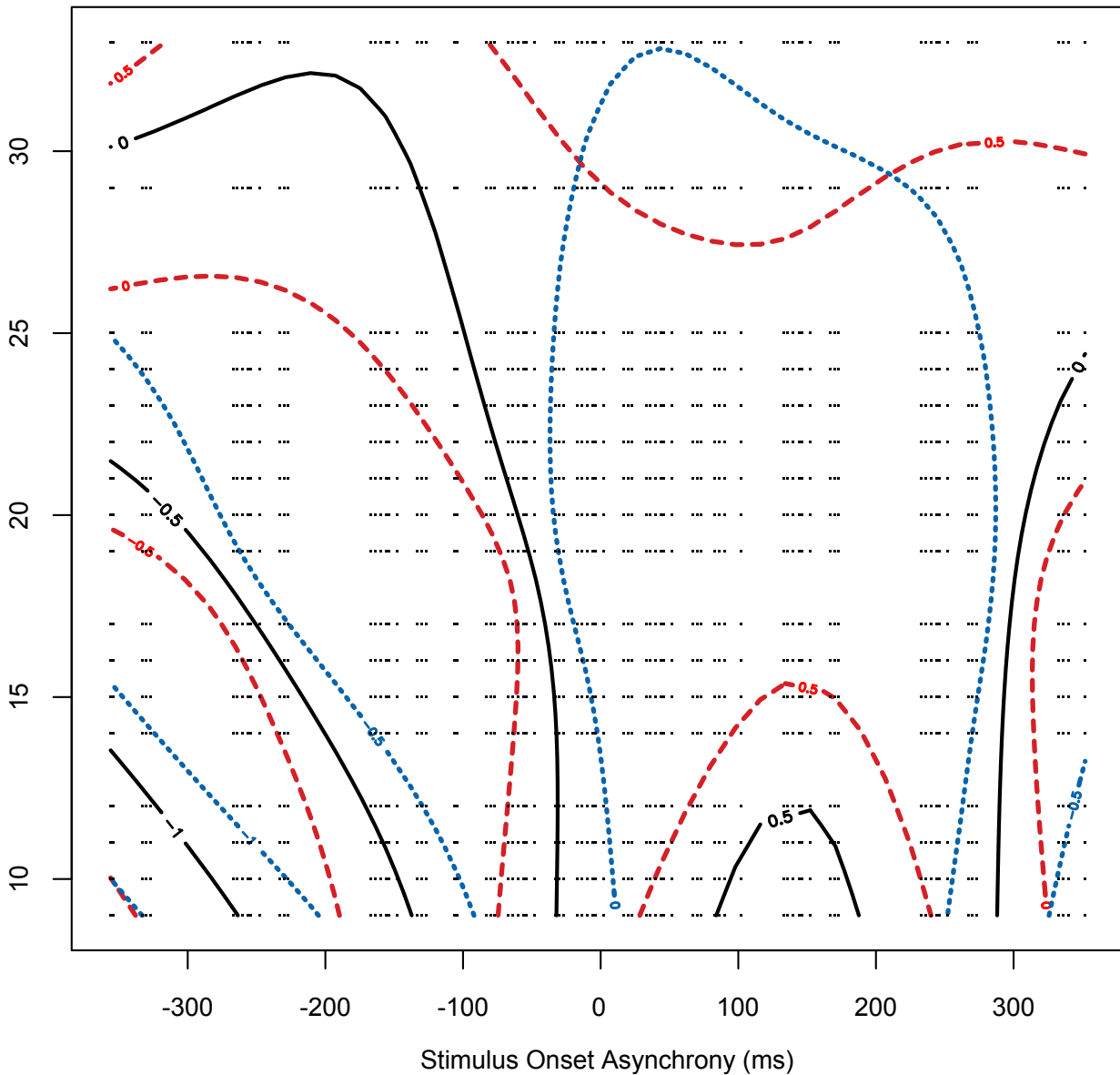

Supplement: 2_SOA_SE.pdf [file NIHMS1725297-supplement-2_SOA_SE_pdf.pdf]

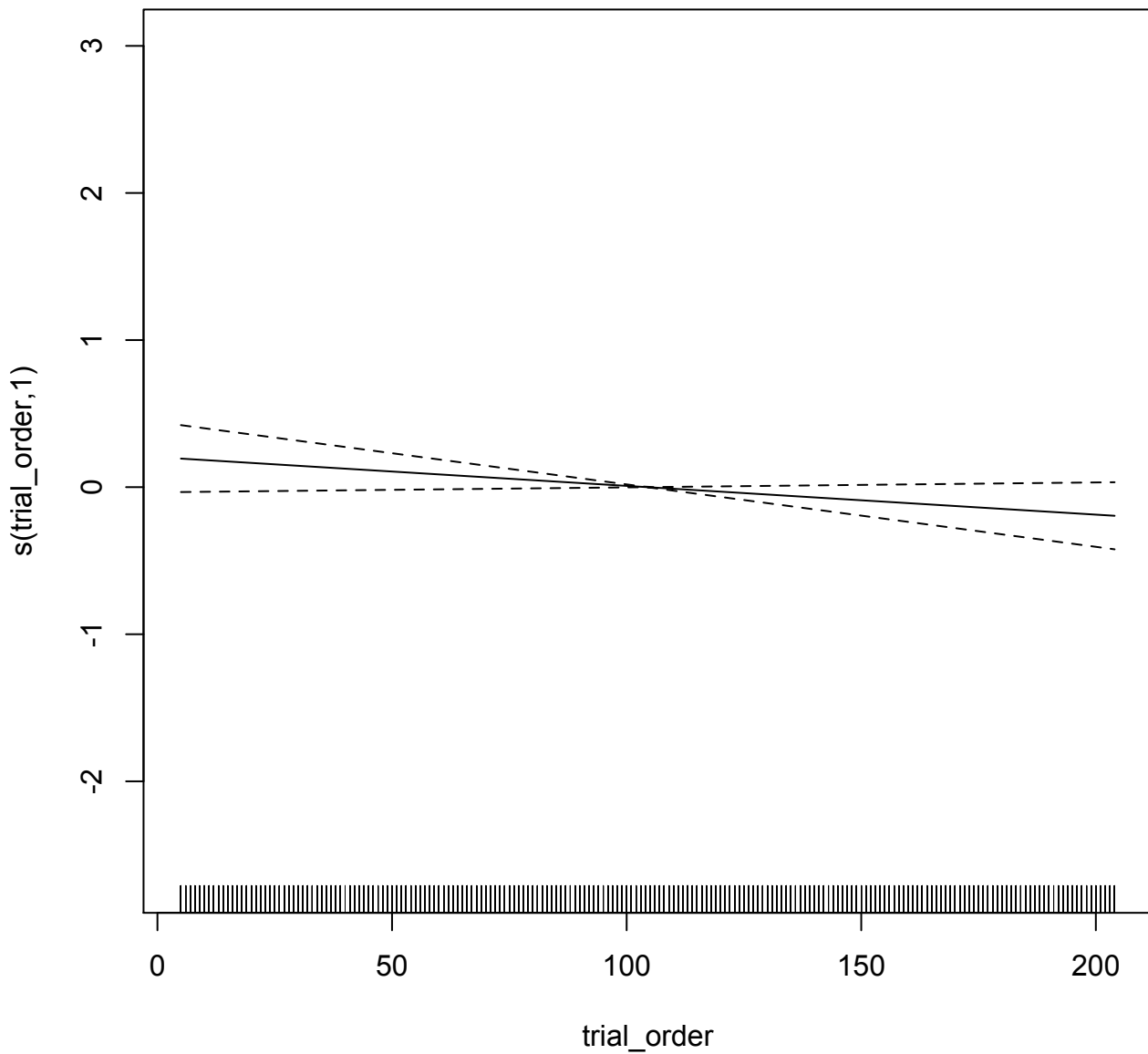

Supplement: 5_trial_order.pdf [file NIHMS1725297-supplement-5_trial_order_pdf.pdf]

**s(mSOA,subject,7.96)**

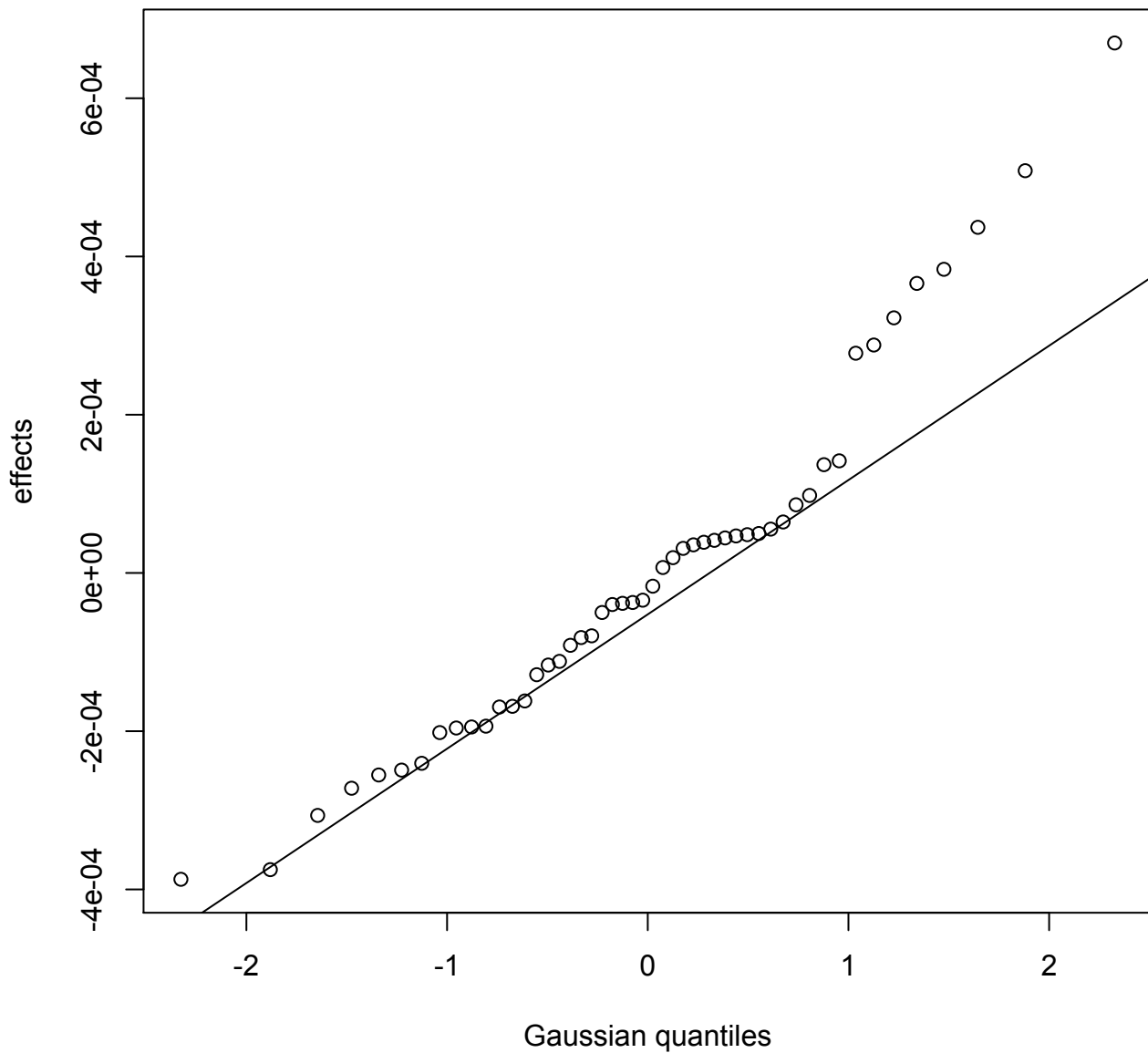

Supplement: 6_SOA_subject_quantile.pdf [file NIHMS1725297-supplement-6_SOA_subject_quantile_pdf.pdf]

**s(AQ\_score,subject,0.02)**

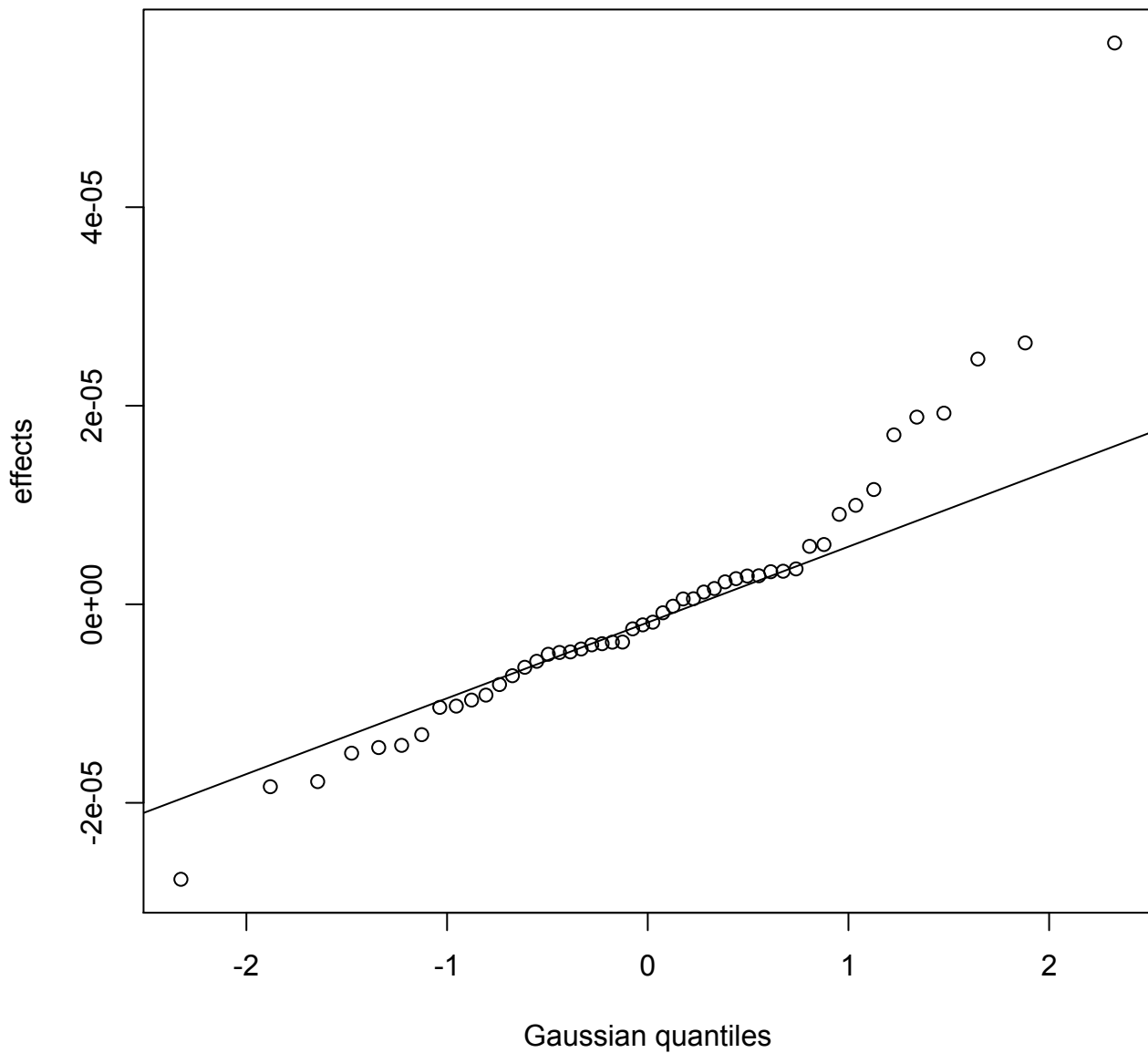

Supplement: 7_AQ_subject_quantile.pdf [file NIHMS1725297-supplement-7_AQ_subject_quantile_pdf.pdf]

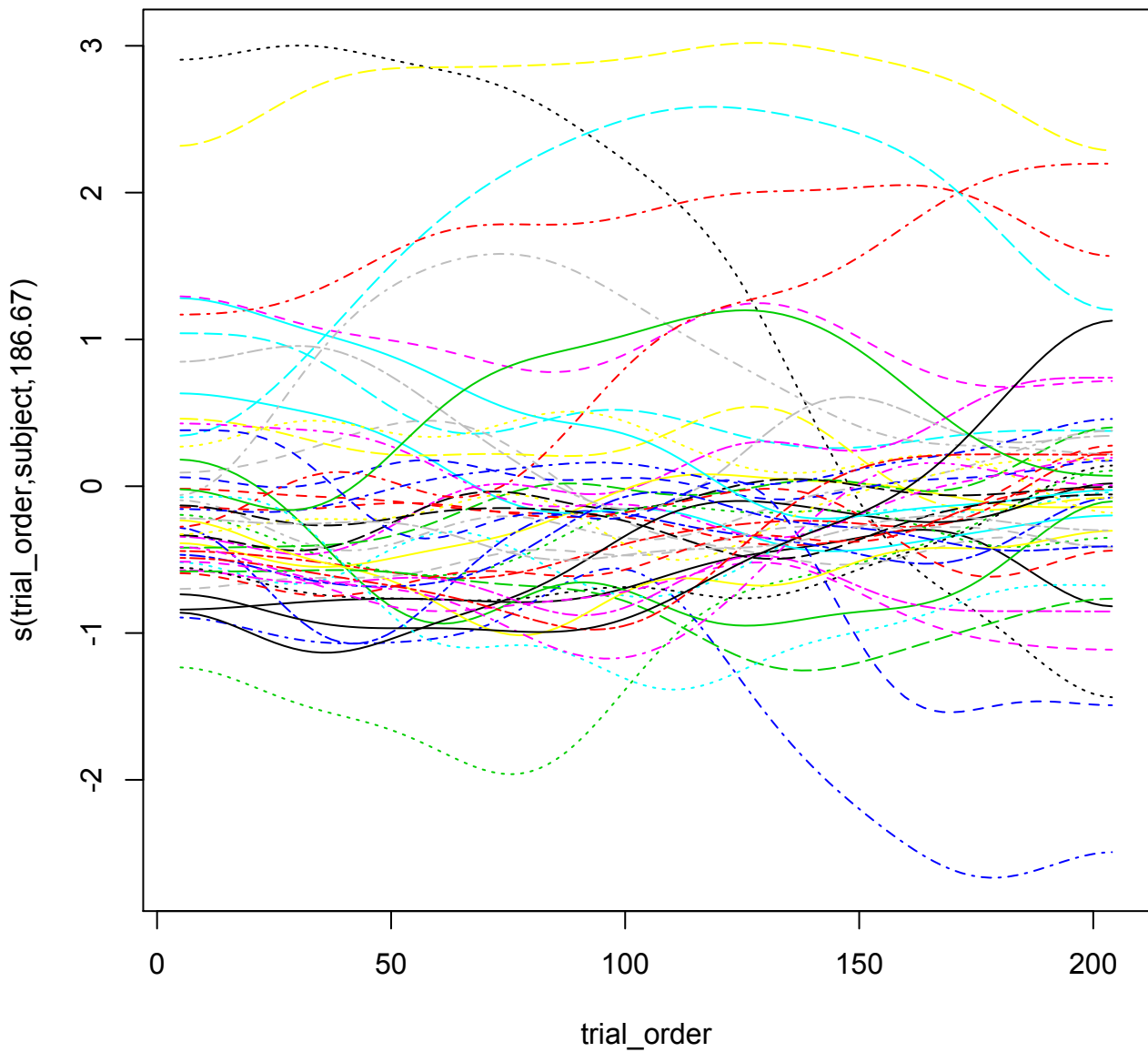

Supplement: 8_trial_order_subject.pdf [file NIHMS1725297-supplement-8_trial_order_subject_pdf.pdf]

**s(movie\_name,8.7)**

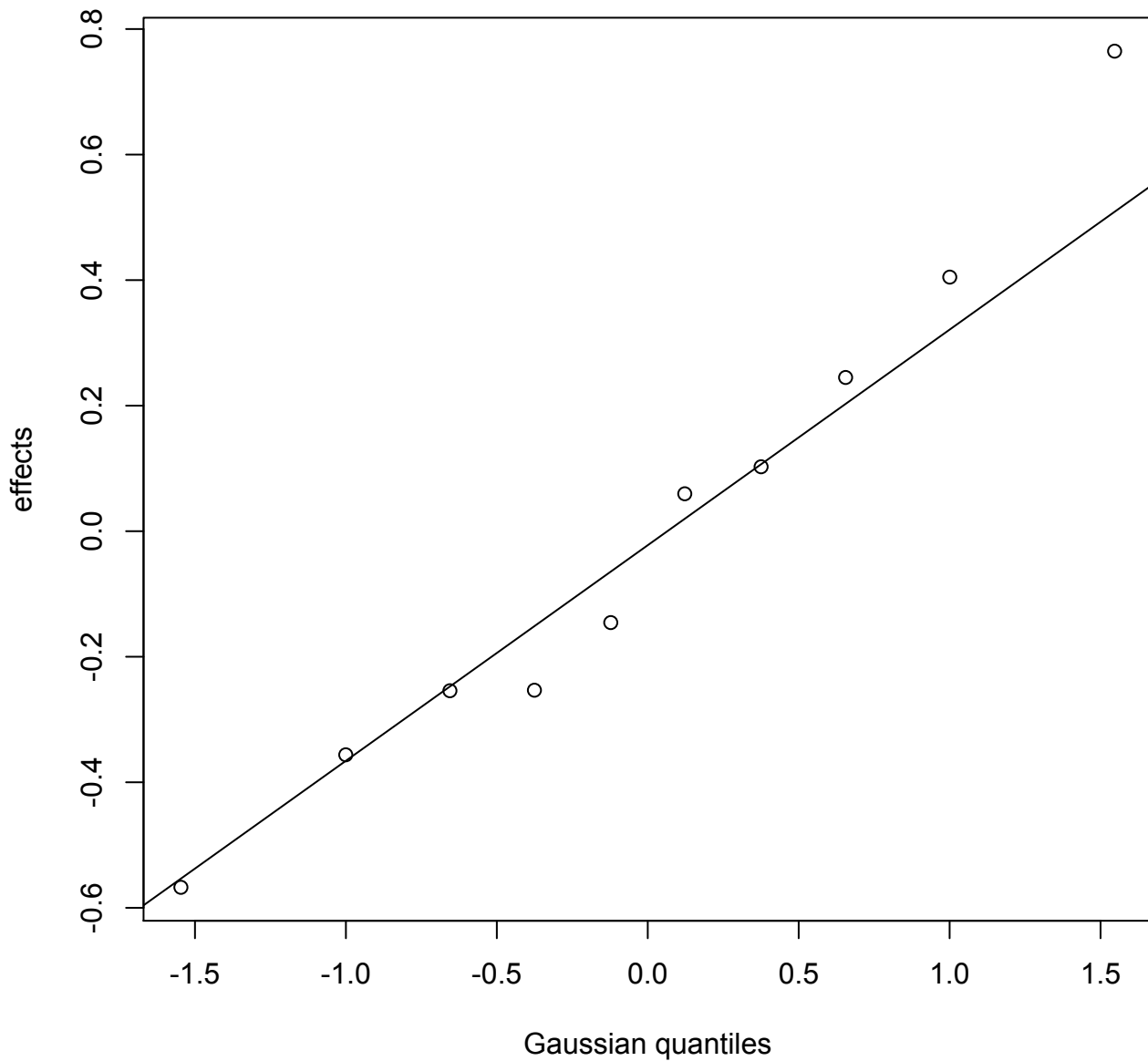

Supplement: 9_token_quantile.pdf [file NIHMS1725297-supplement-9_token_quantile_pdf.pdf]

# ACF resid\_gam(test)

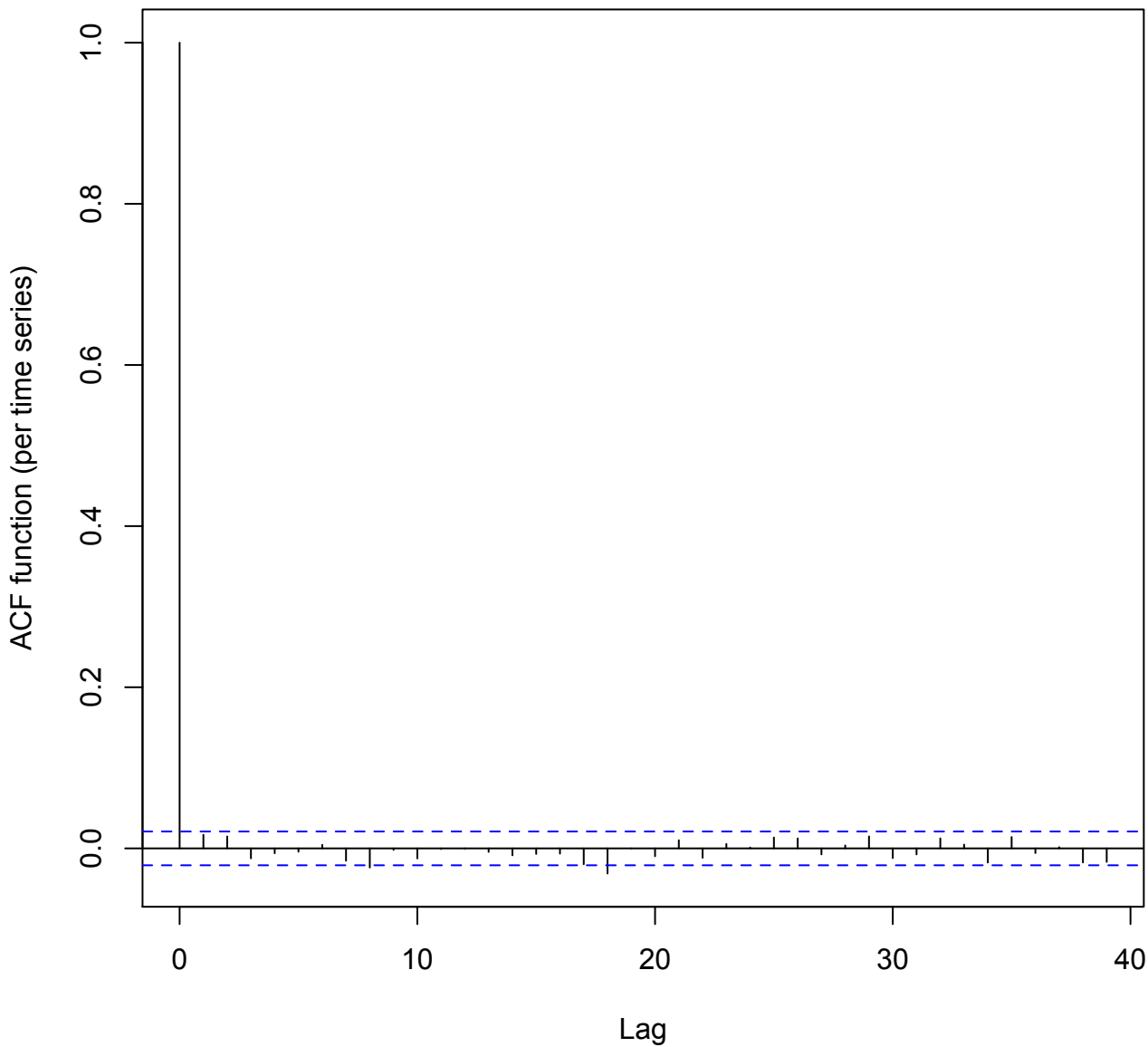

Supplement: 10_ACF.pdf [file NIHMS1725297-supplement-10_ACF_pdf.pdf]
