# Supplementary material for: Visual-Tactile Speech Perception and the Autism Quotient
Source: Front Commun (Lausanne). Author manuscript; Available in PMC 2022 Jan 31. (PMC8802876; doi:10.3389/fcomm.2018.00061)
Supplement: 12_ACF_subject_2.pdf [file NIHMS1725297-supplement-12_ACF_subject_2_pdf.pdf]

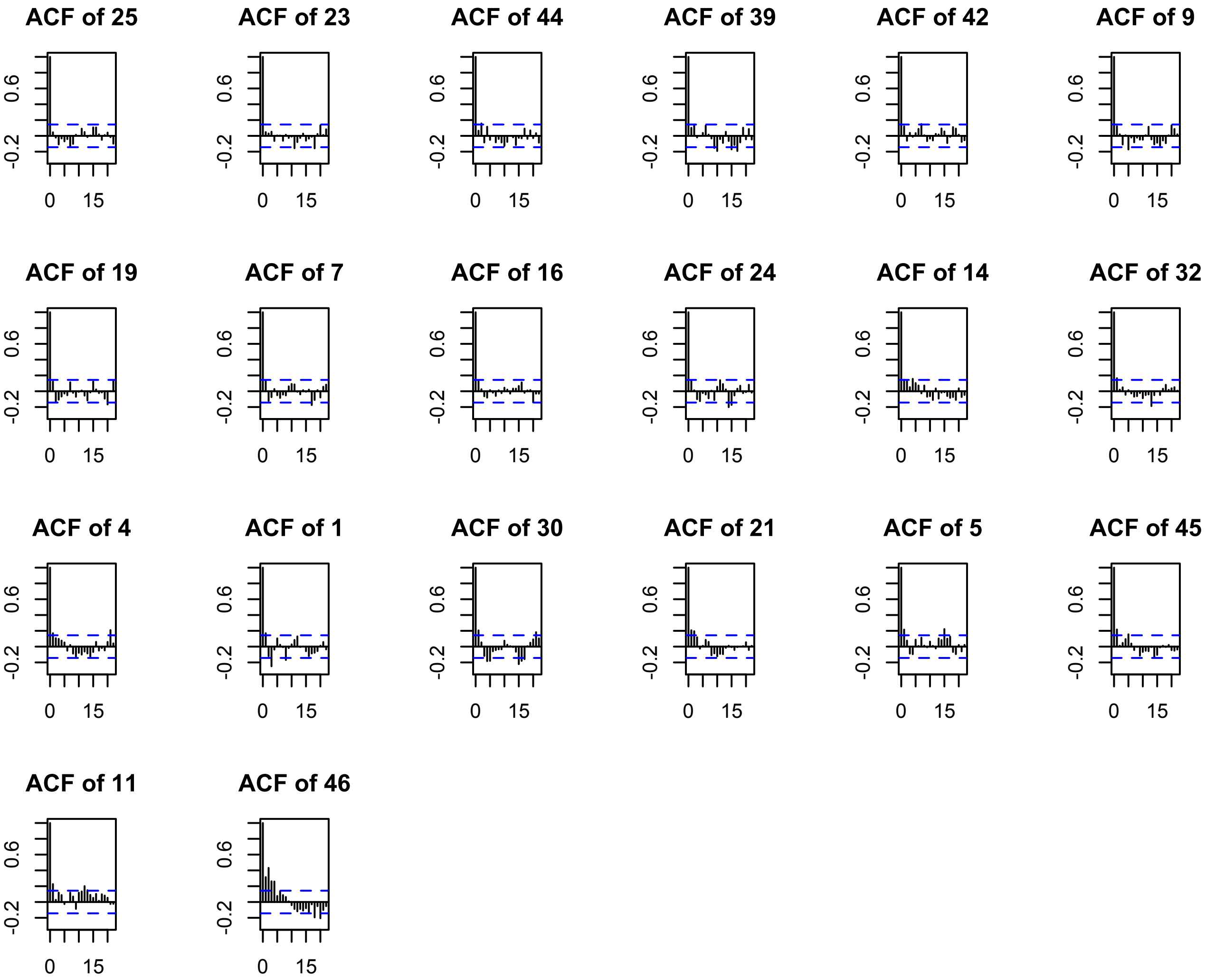
ACF of 25
ACF of 23
ACF of 44
ACF of 39
ACF of 42
ACF of 9
ACF of 19
ACF of 7
ACF of 16
ACF of 24
ACF of 14
ACF of 32
ACF of 4
ACF of 1
ACF of 30
ACF of 21
ACF of 5
ACF of 45
ACF of 11
ACF of 46
0.6
-0.2
0
15
