# Supplementary material for: Visual-Tactile Speech Perception and the Autism Quotient
Source: Front Commun (Lausanne). Author manuscript; Available in PMC 2022 Jan 31. (PMC8802876; doi:10.3389/fcomm.2018.00061)
Supplement: Guide to Supplementary Materials [file NIHMS1725297-supplement-Guide_to_Supplementary_Materials.docx]

**Supplemental materials:**

**Visual-tactile Speech Perception and the Autism Quotient**

Here is provided supplemental materials for the article *Visual-tactile Speech Perception and the Autism Quotient.* These consist of the data from each participant who successfully completed the experiment. Originally, fifty-five people were recorded. Five (5) participants were excluded due to experiment error: One participant altered the volume, the compressor was not turned on for two participants, and babble was not turned on for two more, leaving fifty (50) participants. The supplemental files contain the successful generalized additive mixed-effects models (GAMM), along with all the relevant output graphs and text. These files provide all of the evidence used to show that the selected GAMMs were the most appropriate and explanatory models for this research data.

**Data file**

The data is stored in a CSV file with the headers

**SOA**: {-300,-200.-100,-50,0,50,100,200,300,NA}

Stimulous Onset Asynchrony (measured from the vowel onset)

**puff_condition:** {puff, noPuff}

**movie:** Audio/video file played: controlled the air pump and presented face stimuli

The encoding contains information duplicated in the other fields.

**visual_stim:** {ba, pa}

Visual stimuli from spoken “ba” or “pa”.

**prac_response:** {z, slash}

Recordings of practice responses (first 4 responses)

**trial_response:** {z, slash}

Recordings for trial responses (after the first 4)

**expName:** {VT}

All tokens are VT - the “visual tactile” experiment.

**participant:** {N}

Participant number

**gender:** {m, f}

In this study, all the participants identified as male or female.

**age:** {N}

Age in whole number years

**L1:** {English, Korean …}

Native language

**english_age:** {N}

The age in years when the participant first learned English.

**trial_order:** {N}

The trial order of token presentation.

**movie_name:** {pa{1-5}, ba{1-5}}

The underlying video file played to the participant.

**AQ_score:** {N}

Autism quotient score.

**SS:** {N}

Social skill portion of the Autism Quotient.

**AS:** {N}

Attention switching portion of the Autism Quotient.

**I:** {N}

Imagination portion of the Autism Quotient.

**AD:** {N}

Attention to detail portion of the Autism Quotient.

**C:** {N}

Communication portion of the Autism Quotient.

**Graphs and output files**

The graphs in the supplemental materials can be produced by running

1_SOA_AQ.pdf

The underlying topographical graph of the stimulus onset asynchrony and autism quotient used in the main article.

2_SOA_SE.pdf

The underlying standard error graph of the stimulus onset asynchrony and autism quotient used in the main article.

3_SOA.pdf

A graph of the overall effect of SOA (as measured from the burst onset) on whether people identify “pa” or “ba”. It shows that for all the data, perceivers identify the stimuli as “pa” when the SOA’s closer to 0.

4_AQ.pdf

The linear effect of autism quotient on peoples’ perception of the stimuli. There is no significant overall effect of AQ on whether perceivers identify the stimuli as “pa” or “ba” – the effects are all related to the shape of the windows of integration.

5_trial_order.pdf

The linear effect of trial order on peoples’ perception of the stimuli. There is no significant overall effect of trial on whether perceivers identify the stimuli as “pa” or “ba” – the effects are all related changes in attention during the experiment.

6_SOA_subject_quantile.pdf

Quantiles related to SOA and participant.

7_AQ_subject_quantile.pdf

Quantiles related to Autism Quotient and participant.

8_trial_order_subject.pdf

This graphs shows the effect of trial order on perceptual response – it shows the non-linear nature of attention during experiments.

9_token_quantile.pdf

Quantiles related to the individual tokens (which of the 10 movies was played)

10_ACF.pdf

Autocorrelation function for the whole dataset.

11_ACF_subject_1.pdf

12_ACF_subject_2.pdf

Autocorrelation functions for each subject. There were some participants with higher-than ideal autocorrelations (especially for subject 46), but to be expected for a dataset of this kind.

all_participants_AQ_summary.csv

Contains the experimental data from all of the 50 participants.

SOA_models_GAMM_AQ.R

A r-script, dependent on the mgcv and itsadug, that produces Generalized Additive Mixed-effects models for the relationship between SOA and AQ (one version for SOA based on vowel onset, and one version for SOA based on release burst). This file contains all the tests referenced in the paper.

output_GAMM.txt

A file containing the statistical output for SOA_models_GAMM_AQ.R
