# Supplementary material for: Visual-Tactile Speech Perception and the Autism Quotient
Source: Front Commun (Lausanne). Author manuscript; Available in PMC 2022 Jan 31. (PMC8802876; doi:10.3389/fcomm.2018.00061)
Supplement: 11_ACF_subject_1.pdf [file NIHMS1725297-supplement-11_ACF_subject_1_pdf.pdf]

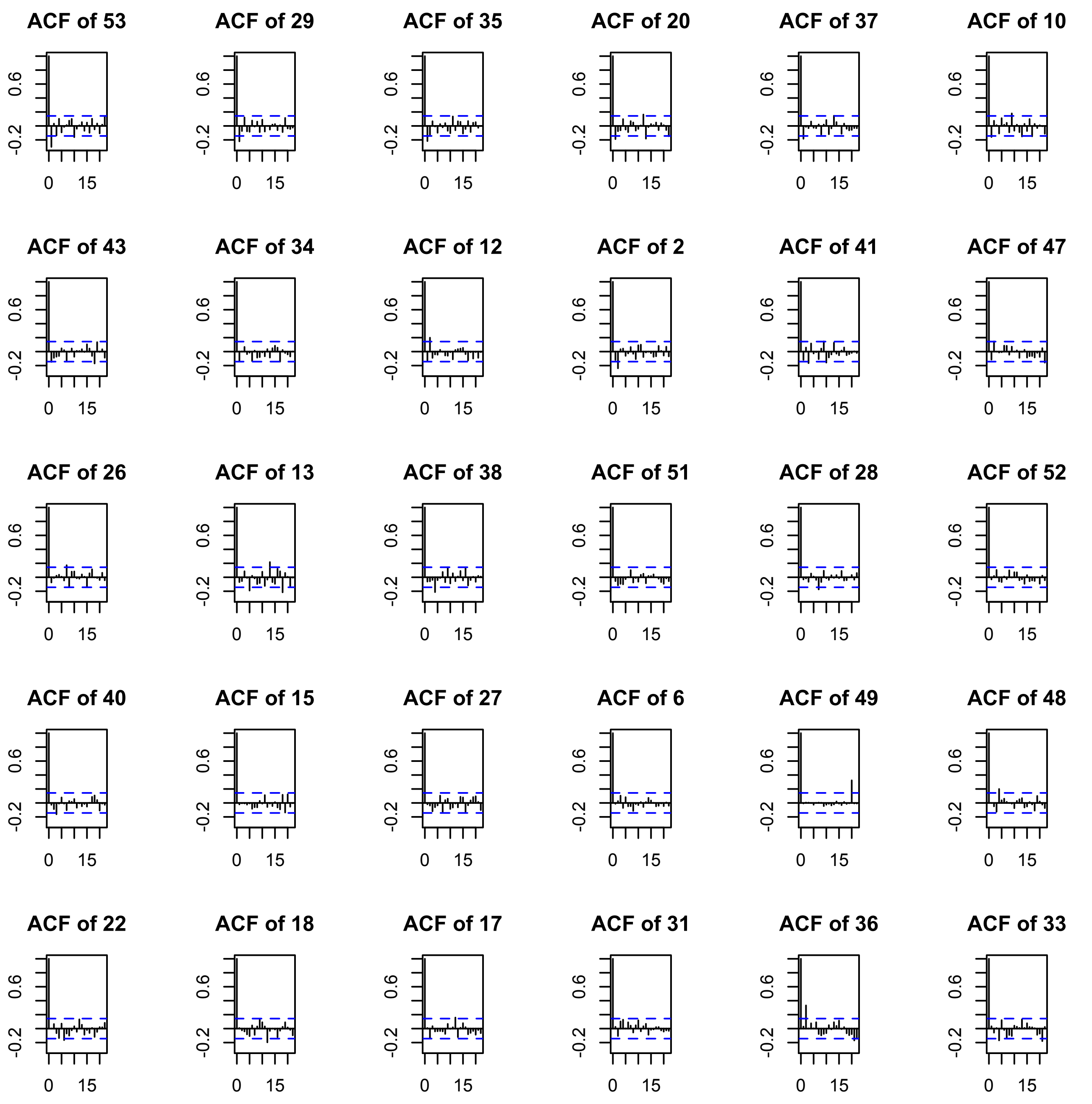
ACF of 53
ACF of 29
ACF of 35
ACF of 20
ACF of 37
ACF of 10
ACF of 43
ACF of 34
ACF of 12
ACF of 2
ACF of 41
ACF of 47
ACF of 26
ACF of 13
ACF of 38
ACF of 51
ACF of 28
ACF of 52
ACF of 40
ACF of 15
ACF of 27
ACF of 6
ACF of 49
ACF of 48
ACF of 22
ACF of 18
ACF of 17
ACF of 31
ACF of 36
ACF of 33
0.6
-0.2
0
15
